# Prehospital Lyophilized Plasma Transfusion for Trauma-Induced Coagulopathy in Patients at Risk for Hemorrhagic Shock: A Randomized Clinical Trial

**DOI:** 10.1001/jamanetworkopen.2022.23619

**Published:** 2022-07-26

**Authors:** Daniel Jost, Sabine Lemoine, Frédéric Lemoine, Clément Derkenne, Sébastien Beaume, Vincent Lanoë, Olga Maurin, Emilie Louis-Delaurière, Maëlle Delacote, Pascal Dang-Minh, Marilyn Franchin-Frattini, René Bihannic, Dominique Savary, Albrice Levrat, Clémence Baudouin, Julie Trichereau, Marina Salomé, Benoit Frattini, Vivien Hong Tuan Ha, Romain Jouffroy, Edouard Seguineau, Rudy Titreville, Florian Roquet, Olivier Stibbe, Benoit Vivien, Catherine Verret, Michel Bignand, Stéphane Travers, Christophe Martinaud, Michel Arock, Mathieu Raux, Bertrand Prunet, Sylvain Ausset, Anne Sailliol, Jean-Pierre Tourtier

**Affiliations:** 1 Paris Fire Brigade Medical Emergency Department, Paris, France; 2 Bataillon de Marins-Pompiers de Marseille, Marseille, France; 3 Direction de la Formation, de la Recherche et de l’Innovation, Service de Santé des Armées, Paris, France; 4 Emergency Department, Angers University Hospital, Angers, France; 5 Research Institute for Environmental and Occupational Health–Unité Mixte de Recherche (UMR)_S 1085, France Emergency Department, Angers University Hospital, Angers, France; 6 Department of Intensive Care, Annecy Hospital, Annecy, France; 7 Service Mobile d’Urgence et de Réanimation de Paris, Hôpital Lariboisière, Assistance Publique–Hôpitaux de Paris (AP-HP) and Université de Paris, Paris, France; 8 Intensive Care Unit, Ambroise Paré Hospital, AP-HP and Paris Saclay University, Boulogne Billancourt, France; 9 Service d’Anesthésie-Réanimation, Hôpital Européen Georges-Pompidou, AP-HP, Paris, France; 10 Service de Biostatistique et Informatique Médicale, Unité Institut National de la Santé et de la Recherche Médicale, UMR 1153, Université Paris Diderot, Paris, France; 11 Service d’Aide Médicale Urgente de Paris, Hôpital Necker-Enfants Malades, AP-HP and Université de Paris, Paris, France; 12 Department of Clinical Operations, French Military Blood Institute, Clamart, France; 13 Laboratory of Hematology, Pitié-Salpêtrière Hospital, Paris, France; 14 Département d’Anesthésie Réanimation, AP-HP Groupe Hospitalier Universitaire, AP-HP–Sorbonne Université, Site Pitié-Salpêtrière, Paris, France; 15 Department of Anesthesiology and Intensive Care, Percy Military Teaching Hospital, Clamart, France; 16 Department of Anesthesiology and Intensive Care, Bégin Military Teaching Hospital, Saint-Mandé, France

## Abstract

**Importance:**

Blood transfusion is a mainstay of therapy for trauma-induced coagulopathy, but the optimal modalities for plasma transfusion in the prehospital setting remain to be defined.

**Objective:**

To determine whether lyophilized plasma transfusion can reduce the incidence of trauma-induced coagulopathy compared with standard care consisting of normal saline infusion.

**Design, Setting, and Participants:**

This randomized clinical trial was performed at multiple centers in France involving prehospital medical teams. Participants included 150 adults with trauma who were at risk for hemorrhagic shock and associated coagulopathy between April 1, 2016, and September 30, 2019, with a 28-day follow-up. Data were analyzed from November 1, 2019, to July 1, 2020.

**Intervention:**

Patients were randomized in a 1:1 ratio to receive either plasma or standard care with normal saline infusion (control).

**Main Outcomes and Measures:**

The primary outcome was the international normalized ratio (INR) on arrival at the hospital. Secondary outcomes included the need for massive transfusion and 30-day survival. As a safety outcome, prespecified adverse events included thrombosis, transfusion-related acute lung injury, and transfusion-associated circulatory overload.

**Results:**

Among 150 randomized patients, 134 were included in the analysis (median age, 34 [IQR, 26-49] years; 110 men [82.1%]), with 68 in the plasma group and 66 in the control group. Median INR values were 1.21 (IQR, 1.12-1.49) in the plasma group and 1.20 (IQR, 1.10-1.39) in the control group (median difference, −0.01 [IQR, −0.09 to 0.08]; *P* = .88). The groups did not differ significantly in the need for massive transfusion (7 [10.3%] vs 4 [6.1%]; relative risk, 1.78 [95% CI, 0.42-8.68]; *P* = .37) or 30-day survival (hazard ratio for death, 1.07 [95% CI, 0.44-2.61]; *P* = .89). In the full intention-to-treat population (n = 150), the groups did not differ in the rates of any of the prespecified adverse events.

**Conclusions and Relevance:**

In this randomized clinical trial including severely injured patients at risk for hemorrhagic shock and associated coagulopathy, prehospital transfusion of lyophilized plasma was not associated with significant differences in INR values vs standard care with normal saline infusion. Nevertheless, these findings show that lyophilized plasma transfusion is a feasible and safe procedure for this patient population.

**Trial Registration:**

ClinicalTrials.gov Identifier: NCT02736812

## Key Points

**Question:**

Does prehospital transfusion of lyophilized plasma result in a lower incidence of trauma-induced coagulopathy at hospital admission compared with standard care with normal saline infusion in patients at risk for hemorrhagic shock after trauma?

**Findings:**

This multicenter randomized clinical trial included 150 patients with trauma who were treated in a prehospital setting. Median international normalized ratio at hospital admission, massive transfusion rate, 30-day survival, and adverse events did not significantly differ between lyophilized plasma transfusion and standard care.

**Meaning:**

These findings suggest that prehospital lyophilized plasma transfusion is a feasible and safe procedure for patients who are at risk for hemorrhagic shock, although there is a lack of evidence regarding its ability to prevent trauma-induced coagulopathy.

## Teaser

This multicenter randomized clinical trial assesses whether prehospital use of lyophilized plasma compared with normal saline infusion by advanced life support teams decreases the incidence of trauma-induced coagulopathy among patients who are risk for hemorrhagic shock.

## Introduction

Trauma-induced coagulopathy (TIC) is an abnormal coagulation status attributed to trauma that affects 20% to 50% of patients with severe trauma.^1,2^ Its occurrence increases transfusion requirements, multivisceral failure, and mortality.^3^ The underlying process involves endothelial, inflammatory, and immunological mediators.^4^ Exogenous factors, such as hemodilution and hypothermia, aggravate the condition and require substitution treatment with labile blood products.^3,5,6^ After publication of findings from the COMBAT (Control of Major Bleeding After Trauma)^7^ and PAMPer (Prehospital Air Medical Plasma)^8^ trials, evidence supports a potential mortality benefit of prehospital fresh frozen plasma for patients with blunt injuries, transport time exceeding 20 minutes, severe shock with higher lactate levels, traumatic brain injury, and moderate transfusion requirements.

Freeze-dried plasma has been used in military settings for several decades and has emerged as an alternative owing to the logistical, storage, resource, ABO compatibility, and cost constraints associated with fresh-frozen plasma.^6,7,8,9^ In the Prehospital Lyophilized Plasma (PREHO-PLYO) trial, we hypothesized that prehospital use of lyophilized plasma by advanced life support (ALS) teams would decrease TIC incidence in patients with trauma at risk for hemorrhagic shock compared with standard care with normal saline infusion.

## Methods

### Study Design and Participants

The PREHO-PLYO trial was an open-label, randomized clinical trial conducted with physician-staffed ALS teams. The study design has been described previously.^10^ Eligible individuals were patients with severe trauma 18 years or older at high risk for hemorrhagic shock and associated coagulopathy whose initial care was managed by ALS teams during ground transportation to a level 1 trauma center (eTable 1 in Supplement 1). Prehospital physicians assessed severity using clinical, circumstantial, and Vittel kinetic criteria (physiological variables, anatomical injuries, prehospital resuscitation, patient predisposition, and kinetic components) that are close to the 2011 guidelines for field triage and commonly used in the French emergency medical response system.^11,12^ The risk of coagulopathy was considered high with a first observation of systolic blood pressure less than 70 mm Hg or a shock index (calculated as heart rate divided by systolic blood pressure) greater than 1.1.^13^ Exclusion criteria were age younger than 18 years, refusal to participate, liberty deprivation, pregnancy, known allergy to amotosalen, prehospital administration of coagulation factors, initial cardiac arrest, or a known do-not-resuscitate status from the prehospital setting. We collected individual consent before inclusion if the patient’s state of consciousness allowed it. In cases of unconsciousness, the physician provided the allocated treatment in the patient’s best interest and collected deferred consent as required by French law.^14^

We conducted this study in accordance with the Declaration of Helsinki,^15^ French law, and good clinical practice. The Ethics Committee of Ile-de-France III approved the trial protocol (Supplement 2) on November 17, 2015. The study followed the Consolidated Standards of Reporting Trials (CONSORT) reporting guideline.

### Randomization and Treatment Allocation

We used individual randomization based on a 1:1 allocation ratio. The French Military Blood Institute prepared, numbered, stored, and delivered the study bags in pairs to the ambulances, with each bag containing either 4 lyophilized plasma units (200 mL each) and the water necessary for its reconstitution, or 2 normal saline packs (500 mL each). At the time of inclusion, the ALS teams had to unseal the bag labeled with the number that the dispatcher had transmitted.

### Procedures

The French emergency medical services system is a 2-tiered response system.^16^ Basic life support ambulances, which are crewed by 3 firefighters, have a network density that allows them to be dispatched as precursors to individuals who are experiencing trauma. An ALS team with an emergency physician is dispatched immediately after the dispatch center identifies qualifying severity criteria (Vittel criteria).^12^ Basic life support teams, which in most cases are the first to arrive at a scene, perform requisite life-saving actions. The ALS relay teams assess the patient’s eligibility and draw the first blood sample using a point-of-care coagulometer (Coaguchek Pro II System; Roche Diagnostics) to acquire an international normalized ratio (INR) measurement at the point of injury. The physician subsequently administers the allocated treatment by transfusing as many as 4 U of plasma (800 mL) or infusing as many as 1000 mL of crystalloid, depending on the group allocation. In our study, regardless of the study group, the physician had the flexibility to adapt the normal saline volumes that were administered to achieve hemodynamic goals following the guidelines for posttraumatic hemorrhagic shock (eFigure 1 in Supplement 1). During the transfer, the ALS team measured a second INR using the point-of-care coagulometer. At hospital arrival, the trauma team performed blood tests (INR, prothrombin time, and levels of fibrinogen and factors II, V, VII, and X) before administering any inhospital transfusion. Viscoelastic testing was not routinely available in the participating hospitals at the time of the study.

### Outcomes and Variables

The primary outcome was the INR value at hospital admission, which represents the most commonly and easily performed coagulation test to assess TIC in our system. The INR thresholds used to diagnose TIC vary from study to study.^5,13^ In this trial, we used an INR threshold value of 1.2.

Secondary outcomes were the INR difference between the 2 prehospital point-of-care measures (ΔINR); the need for massive blood transfusion predefined as the transfusion of more than 10 U of red blood cells within 24 hours after injury; the need for hospital blood-component transfusions at early time points (6-24 hours); fibrinogen level at hospital arrival; surgical procedures in the first 24 hours; days free of ventilator use or the intensive care unit; incidence of complications (multiorgan failure, kidney failure, sepsis, thrombosis); and 30-day survival.^17^ Additional variables related to demographics, time frames, severity of the patient’s condition, prehospital resuscitation, biological parameters (levels of lactate, hemoglobin, and base excess), fluid volumes, and plasma-to-crystalloid volume ratio (volume of lyophilized plasma divided by the volume of normal saline administered).^18,19^

### Usability

Usability end points were the technical and logistical difficulties encountered during plasma transfusion. We collected them via predefined items and open declarative data from the physician. Furthermore, we expected that the plasma preparation time would not extend the prehospital care time.

### Safety

An independent data monitoring committee oversaw safety. Safety outcomes were the occurrence of prespecified adverse events: thrombosis, allergic symptoms, transfusion-related acute lung injury, and transfusion-associated circulatory overload. We assessed the traceability of plasma, including prescription, delivery, and follow-up procedures, using the plasma follow-up sheet from the French Military Blood Institute.

### Statistical Analysis

Data were analyzed from November 1, 2019, to July 1, 2020. We finalized the statistical analysis plan before the database lock (Supplement 2). In the first protocol version, the primary end point was the ΔINR. However, at the statistical analysis planning stage, we changed the end point to the INR at hospital arrival, because prehospital constraints for blood drawing became prominent. We considered ΔINR a secondary outcome and calculated it for patients with available blood samples (eFigure 2 in Supplement 1). Based on this amended primary outcome, a sample size of 30 patients with coagulopathy per group was adequate to detect a mean difference in the INR at hospital admission of 0.3, assuming an SD of 0.4, with a 2-sided type I error of .05 and power of 80%. Unpublished data from a previous study also support this effect size.^20^ This requirement results in 60 patients per group with an assumed incidence of 50% for coagulopathy. Given the a priori loss of 10% due to lack of informed consent, we rounded the target size to 70 patients per group. To consider a median ΔINR of 0.30, the sample needed to include approximately 25 patients with coagulopathy per group, based on the same alpha and beta risk assumptions.^21^ We presented the median INR values with their IQRs and based them on a 3-category variable with cutoff values of 1.20 and 1.50.

Twenty months into the study, we broadened the inclusion criteria by lowering the shock index threshold from 1.3 to 1.1 because of recruitment difficulties. We performed a post hoc subanalysis and fit our models by considering a before-and-after dummy variable referring to this threshold change. We reported categorical variables as frequencies and used a χ^2^ test or Fisher exact test in comparisons. Continuous variables are expressed as medians (IQRs) and were compared using a Mann-Whitney *U* test. We handled missing data by performing multiple imputation procedures, including variables under the assumption that missing values were missing at random (eTable 2 and eFigure 3 in Supplement 1).^22^ If significant differences were absent between observed and imputed values, we presented the imputed values. We conducted intention-to-treat analyses for the safety outcome and preplanned modified intention-to-treat analyses excluding patients with trauma-free gastrointestinal tract or obstetric bleeding for the other outcomes. The latter received plasma transfusions based on the physician’s decision to unseal the bags outside a traumatic context. We compared the ΔINR values between the groups using a linear mixed model with an independent covariance matrix considering study group, time, and Injury Severity Score levels as fixed effects and incorporating a random intercept. We described survival times using a Kaplan-Meier diagram and log-rank test. We fit a Cox proportional hazards regression model adjusted for age and Injury Severity Score and reported the effects as hazard ratios with 95% CIs. As exploratory measures, we calculated the treatment effect size in following subgroups: type of injury, presence of pelvic injury, prehospital vasopressor use, prehospital intubation, brain injury with bleeding on computed tomography, massive transfusion, and death after 24 hours. We represented effect sizes by the median difference (95% CI) for continuous variables and risk ratio (95% CI) for categorical variables. We performed all comparisons using 2-sided tests; we considered *P* < .05 to be significant. The statisticians (D.J., F.L., J.T., V.H., F.R., C.V.) were blinded to the patient’s treatment. We conducted analyses with StataH software, version 14.0 (StataCorp LLC).

## Results

**Figure 1.**
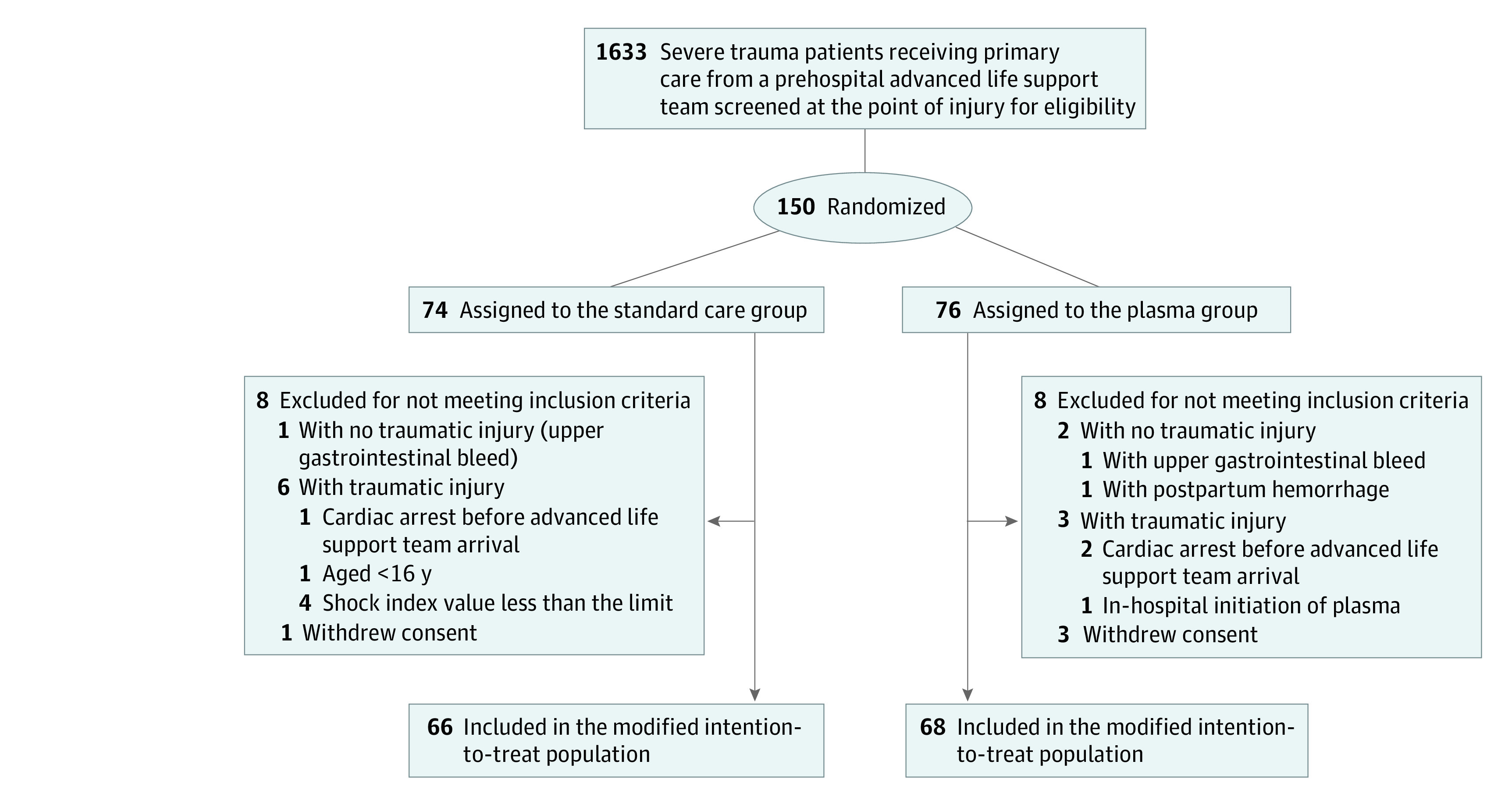
Flowchart of Trial Inclusion The modified intention-to-treat population included all randomized patients, excluding those who were deemed ineligible after randomization. Patients who withdrew consent were not included in the analysis.

From April 1, 2016, to September 30, 2019, we screened 1633 patients treated by ALS teams for inclusion and deemed 150 eligible. We randomly assigned 76 patients to the plasma group and 74 to the control group. Sixteen patients met the exclusion criteria. Thus, 134 patients constituted the modified intention-to-treat cohort (68 in the plasma group and 66 in the control group) (Figure 1). The median patient age was 34 (IQR, 26-49) years; 110 (82.1%) were men and 24 (17.9%) were women. Race and ethnicity data were not collected. Most patients had blunt trauma (80 [59.7%]). Demographic characteristics, response times, vital signs at ALS arrival, injury characteristics, and the rates of red blood cell transfusion from the prehospital phase were similar between the 2 groups (Table 1). The median crystalloid volume was 700 (IQR, 475-1000) mL in the plasma group and 1000 (IQR, 700-1350) mL in the control group (*P* = .03). The median volume of plasma was 525 (IQR, 350-800) mL, with a median plasma-to-crystalloid volume ratio of 0.23 (IQR, 0.17-0.40) with 1 U of plasma and 1.60 (IQR, 0.75-2.67) with 4 U of plasma (eFigure 4 in Supplement 1). The median time from ALS team arrival at the point of injury to hospital admission was 62 (IQR, 50-72) minutes in the plasma group and 61 (IQR, 50-75) minutes in the control group (*P* = .78). Patient characteristics were similar before and after we revised the shock index threshold (eTable 3 in Supplement 1).

**Table 1.** Baseline Characteristics

Characteristic	Treatment group^a^	
	Control group (n = 66)	Plasma group (n = 68)
**Prehospital arrival at point of injury**		
Demographics		
Age, median (IQR), y	33.6 (25.2-47.6)	36.6 (26.8-49.5)
Sex		
Men	51 (77.3)	59 (86.8)
Women	15 (22.7)	9 (13.2)
Comorbidities present	6 (9.1)	12 (17.6)
Pathology affecting hemostasis^b^	1 (1.5)	6 (8.8)
Type of injury		
Blunt	40 (60.6)	40 (58.8)
Penetrating	26 (39.4)	28 (41.2)
Mechanism of injury		
Fall	16 (24.2)	18 (26.5)
Stab wound	17 (25.7)	23 (33.8)
Firearm	5 (7.6)	1 (1.5)
Motor vehicle crash	24 (36.4)	22 (32.3)
Other	4 (6.1)	4 (5.9)
**Therapeutics at point of injury**		
Tourniquet use (BLS team)	9 (13.6)	2 (2.9)
Hemostatic dressing use (BLS team)	31 (47.0)	31 (45.6)
Time of response of ALS teams, median (IQR), min		
From call to arrival at the point of injury^c^	23 (15-33)	25 (15-36)
From arrival at the point of injury to hospital	61 (50-75)	62 (50-72)
From injury to arrival at hospital	90 (72-102)	91 (70-115)
Vital status (by ALS teams)		
Glasgow Coma Scale score <8^d^	11 (16.7)	10 (14.7)
Shock index, median (IQR)^e^	1.38 (1.22-1.62)	1.40 (1.22-1.71)
Heart rate, median (IQR), beats/min	115 (101-130)	120 (105-140)
Blood pressure, median (IQR), mm Hg		
Systolic	83 (70-93)	80 (65-96)
Diastolic	54 (41-60)	49 (40-49)
Oxygen saturation, ambient air, median (IQR), %	98 (95-99)	97 (94-99)
Body temperature, median (IQR), °C	36.1 (35.5-36.8)	36.3 (35.7-36.8)
Biological measures (by ALS teams)		
Hemoglobin concentration, median (IQR), g/dL	13.3 (11.5-14.7)	13.1 (11.6-14.7)
Lactate concentration, median (IQR), mg/dL	41.4 (29.7-64.0)	40.5 (18.9-62.2)
Therapeutics (by ALS teams)		
Tracheal intubation	16 (24.2)	22 (32.3)
Thoracic tube	4 (6.1)	2 (2.9)
Vasopressors	35 (53.0)	33 (48.5)
Tranexamic acid	60 (90.9)	57 (83.8)
Crystalloid volume, median (IQR), mL	1000 (700-1350)	700 (475-1000)
Lyophilized plasma volume, median (IQR), mL	NA	525 (350-800)
No. of red blood cell units given^f^	4 (6.1)	1 (1.5)
**At hospital arrival**		
Vital signs, median (IQR)		
Shock index	1.0 (0.8-1.1)	1.0 (0.8-1.2)
Heart rate, beats/min	109 (88-122)	111 (95-123)
Blood pressure, mm Hg		
Systolic	107 (90-125)	111 (92-127)
Diastolic	66 (56-80)	67 (52-78)
CT scan		
Injury Severity Score, median (IQR)^g^	25 (9-41)	29 (12-48)
Traumatic brain injury confirmed	7 (10.6)	9 (13.2)
Laboratory values		
Hemoglobin concentration, median (IQR), g/dL	11.9 (9.9-13.0)	10.2 (8.1-11.9)
Median platelet count, ×10^3^/μL	223 (155 to 273)	199 (166 to 236)
Lactate concentration, median (IQR), mg/dL^h^	31.5 (18.9-53.1)	28.8 (13.5-61.3)
Base excess, median (IQR), mEq/L^i^	–3.0 (–7.1 to 0)	0 (–4 to 0)

Abbreviations: ALS, advanced life support; BLS, basic life support; NA, not applicable.

SI conversion factors: To convert base excess to millimoles per liter, multiply by 1.0; hemoglobin to grams per liter, multiply by 10.0; lactate to millimoles per liter, multiply by 0.111; platelets to ×10^9^ per liter, multiply by 1.0.

^a^ There were no significant differences in baseline characteristics between the 2 study groups, except for tourniquet use (*P* = .02), median crystalloid volume (*P* = .03), and laboratory hemoglobin concentration (*P* = .006). Outside these 4 variables, *P* values ranged from .06 to .92. Continuous variables were compared using a Mann-Whitney *U* test; categorical variables were compared using a Fisher exact test. Patients constitute individuals included in the modified intention-to-treat analysis. Unless indicated otherwise, data are expressed as No. (%) of patients. Percentages have been rounded and may not total 100.

^b^ Defined as chronic liver failure (ie, alcoholic cirrhosis, hepatocellular carcinoma) or factor V Leiden deficiency.

^c^ Data were only available for patients included by the Paris, France, investigating centers.

^d^ Scores ranged from 3 to 15, with lower score indicating a reduced level of consciousness.

^e^ Calculated as heart rate divided by systolic blood pressure.

^f^ *P* = .20.

^g^ Scores range from 0 to 75, with higher values representing more severe injury.

^h^ The second lactate level measurement was performed on arrival at the trauma center by the hospital laboratory.

^i^ Data were unavailable for 16 patients in the control group and 27 patients in the plasma group.

The primary outcome was available for 128 (95.5%) of the 134 patients in the intention-to-treat cohort. After we performed multiple imputation, the median INR at hospital arrival was 1.21 (IQR, 1.12-1.49) in the plasma group and 1.20 (IQR, 1.10-1.39) in the control group, before any transfusions were performed in the hospital (median difference, −0.01 [IQR, −0.09 to 0.08]; *P* = .88) (Table 2 and eTable 4 in Supplement 1). The INR was greater than 1.20 in 37 (54.4%) and greater than 1.50 in 18 (26.5%) patients in the plasma group vs 41 (62.1%) and 16 (24.2%), respectively, in the control group (*P* = .51). Exploratory subgroup analysis did not reveal any difference in the INR between both groups (eFigure 5 in Supplement 1).

**Table 2.** Primary and Secondary Outcomes

Outcome^b^	Treatment group^a^		Effect size (95% CI)^c^	*P* value
	Control (n = 66)	Plasma (n = 68)		
Primary				
Laboratory INR, median (IQR)	1.20 (1.10-1.39)	1.21 (1.12-1.49)	–0.01 (–0.09 to 0.08)	.88
Laboratory INR in a given range^d^				
<1.20	26 (39.4)	31 (45.6)	1.29 (0.61 to 2.71)	.51
1.20-1.50	25 (37.9)	19 (27.9)	0.64 (0.29 to 1.40)	
>1.50	15 (22.7)	18 (26.5)	1.12 (0.48 to 2.65)	
Secondary				
Median prothrombin time, % of normal^e^	73 (64-82)	75 (52-83)	1.9 (–6.1 to 9.9)	.65
Fibrinogen level, median (IQR), mg/dL	190 (150-230)	210 (150-250)	19 (–11 to 49)	.22
Factor level, median (IQR), %				
II	74.6 (60.1-85.7)	71.1 (59.1-84.5)	–3.5 (–14.5 to 7.6)	.53
V	72.0 (55.1-85.0)	61.3 (36.8-80.5)	–10.7 (–30.2 to 8.8)	.27
VII	81.3 (73.2-93.3)	71.1 (55.7-88.8)	–10.2 (–23.0 to 2.7)	.12
X	76.9 (59.2-88.5)	74.8 (58.8-90.4)	–2.1 (–16.6 to 12.4)	.77
Point-of-care INR measure 1, median (IQR)^f^	1.00 (1.00-1.10)	1.10 (1.00-1.20)	0.10 (0.06 to 0.14)	<.001
Measure 2^g^	1.00 (1.10-1.30)	1.10 (1.10-1.20)	0 (–0.20 to 0.20)	>.99
ΔINR^h^	0 (0-0.003)	0 (0-0.002)	0 (– 0.003 to 0.002)	.68
Massive transfusion received in first 24 h^i^^,^^j^	4 (6.1)	7 (10.3)	1.78 (0.42 to 8.68)	.37
Total 6-h volume of blood components transfused, median (IQR), U^j^				
Packed red blood cells	4 (2-8)	4 (2-6)	0 (–1.9 to 1.9)	.32
Fresh frozen plasma	4 (3-7)	4 (2-6)	0 (–1.1 to 1.2)	.98
Platelets	1 (1-2)	1 (1-2)	0.99 (0.76 to 1.29)	.94
Total 24-h volume of blood components transfused, median (IQR), U^j^				
Packed red blood cells	4 (2-8)	5 (2-7)	1.0 (–0.9 to 2.9)	.65
Fresh frozen plasma	4 (3-8)	5 (3-8)	1.0 (–1.6 to 3.6)	.98
Platelets	1 (1-2)	1 (1-2)	0 (–0.87 to 0.87)	.96
Vasopressors needed within 24 h	33 (50.0)	34 (50.0)	1.03 (0.49 to 2.15)	.93
Urgent surgery during the initial 24 h^k^	47 (71.2)	49 (72.1)	1.04 (0.46 to 2.37)	.91
Duration of stay in the ICU, median (IQR), d	2 (1-7)	3 (1-9)	1.0 (–1.6 to 3.6)	.45
Ventilator-free time to 28-d follow-up, median (IQR), d^l^	28 (27-28)	28 (28-28)	0 (– 0.22 to 0.22)	.39
Time out of ICU to 28-d follow-up, median (IQR), d^l^	26 (23-27)	25 (17-27)	–1.0 (– 5.9 to 3.9)	.24
Duration of hospitalization, median (IQR), d	10 (2-20)	9 (3-24)	–1.0 (–9 to 7)	.80
Multiorgan failure	3 (4.5)	1 (1.5)	0.31 (0.01 to 4.05)	.29
Sepsis	4 (6.1)	7 (10.3)	1.78 (0.42 to 8.68)	.37
Death				
Within 6 h	2 (3.0)	3 (4.4)	1.48 (0.16 to 18.18)	.67
Within 24 h	6 (9.1)	9 (13.2)	1.52 (0.45 to 5.53)	.45
28-d Mortality	10 (15.2)	12 (17.6)	1.20 (0.43 to 3.37)	.70

Abbreviations: ICU, intensive care unit; INR, international normalized ratio.

SI conversion factor: To convert fibrinogen to grams per liter, multiply by 0.01.

^a^ Unless indicated otherwise, data are expressed as No. (%) of patients. Percentages have been rounded and may not total 100.

^b^ Primary and secondary outcomes refer to the modified intention-to-treat population.

^c^ Effect sizes are represented by the median between-group difference for continuous variables and the unadjusted risk ratio for categorical variables.

^d^ The INR standardizes the prothrombin time results and is calculated as the patient’s prothrombin time divided by the mean normal prothrombin time in the laboratory and raised to a power designated the international sensitivity index. The INR has no unit.

^e^ Expressed in percentage of reference range and calculated by the patient’s prothrombin time divided by the control prothrombin time in the laboratory. The reference range is 70% to 100%.

^f^ The first blood sample was taken by the advance life support teams using a point-of-care coagulometer to measure the INR at the point of injury and before any specific treatment related to the trial.

^g^ The second blood sample was taken by the advance life support teams using a point-of-care coagulometer to measure the INR on arrival at the hospital before any inhospital transfusion.

^h^ Change in prehospital INR levels before and after fluid transfusion. Data were available for 68 patients.

^i^ Defined as more than 10 U of red blood cells in the first 24 hours after hospital arrival.

^j^ The lyophilized plasma administered in the prehospital period was not included in the numbers of blood components transfused.

^k^ Data were unavailable for 17 patients in the control group and 12 in the plasma group.

^l^ Includes 101 surviving patients.

Sixty-eight patients had a ΔINR measurement (33 in the plasma group and 35 in the control group). Their main characteristics did not differ from those of patients without ΔINR measurements (eTable 5 in Supplement 1). The median ΔINR did not differ between the groups (median difference, 0.001 [95% CI, −0.003 to 0.002]; *P* = .68) (Table 2). Seven patients in the plasma group (10.3%) and 4 in the control group (6.1%) required a massive transfusion (relative risk, 1.78 [95% CI, 0.42-8.68]; *P* = .37). Median fibrinogen values at hospital arrival were 210 (IQR, 150-250) mg/dL in the plasma group and 190 (IQR, 150-230) mg/dL in the control group (*P* = .22) (to convert to grams per liter, multiply by 0.01). In the plasma group, an increase in the plasma-to-crystalloid volume ratio was associated with a decrease in INR (odds ratio, 0.71 [95% CI, 0.52-0.97]; *P* = .03) (eTable 6 in Supplement 1).

**Figure 2.**
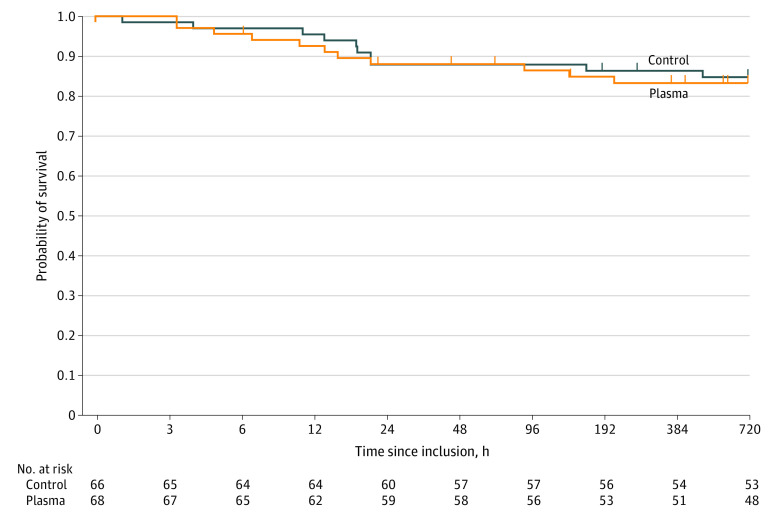
Kaplan-Meier Estimates of Survival at 30 Days A Cox proportional hazards regression model showed no difference in risk of death within 30 days after inclusion between the plasma and control groups (hazard ratio, 1.12 [95% CI, 0.48-2.64]; *P* = .79) or after adjusting for age and Injury Severity Score (hazard ratio, 1.07 [95% CI, 0.44-2.61]; *P* = .89). Tick marks indicate censored data. The time axis is represented on a logarithmic scale.

The 2 groups did not differ in the incidence of surgical procedures in the first 24 hours, incidence of multiorgan failure or sepsis, ventilator-free time, or time out of the intensive care unit (eTable 7 in Supplement 1 and Table 2). Median length of hospital stay was 9 (IQR, 3-24) days in the plasma group and 10 (IQR, 2-20) days in the control group (*P* = .80). Time to death after inclusion was 16 (IQR, 6-96) hours in the plasma group and 21 (IQR, 13-24) hours in the control group (*P* = .60). The 30-day survival probability was 0.83 (IQR, 0.72-0.90) in the plasma group and 0.85 (0.73-0.91) in the control group (*P* = .79) (Figure 2). After adjusting for age and Injury Severity Score, the hazard ratio based on the Cox regression model was 1.07 (95% CI, 0.44-2.61; *P* = .89).

Plasma transfusion was started at a median of 26 (IQR, 16-37) minutes after the ALS team arrived at the point of injury. The time the ALS teams spent on the scene did not differ significantly between the 2 groups (62 [IQR, 50-72] minutes in the plasma group; 61 [IQR, 50-75] minutes in the control group; *P* = .78), indicating a null effect of plasma use on the duration of prehospital care. For 9 of 68 patients in the plasma group, physicians reported either incompletely dissolved residue that obstructed the infusion line or insufficient air admission into the glass vial, resulting in slower transfusion flows (eFigure 6 in Supplement 1).

No transfusion-related events were reported in the prehospital setting, regardless of the number of plasma units transfused (Table 3). In the plasma group, 5 protocol violations were reported compared with 7 in the control group (eTable 8 in Supplement 1). They resulted from noncompliance with one of the inclusion criteria, but without leading to the inclusion of nonbleeding patients.

**Table 3.** Adverse Events Reported in the Intention-to-Treat Population (n = 150)

Adverse events^a^	Treatment group, No. of patients	
	Control (n = 74)	Plasma (n = 76)
All	16	16
Deaths	10	12
Thrombotic events	3	1
Cerebrovascular accident^b^	0	1
Myocardial infarction	1	0
Pulmonary embolism	2	0
Transfusion reaction^c^	0	0
Cardiac arrest	2	0
Sepsis	0	2
Kidney failure	1	0
Seizure	0	1

^a^ Adverse events were defined as any adverse reaction considered to be related to the trial regimen. They were identified and reported at the discretion of the treating physician (investigator) and were all reviewed by the data safety monitoring board (DSMB), which determined causality.

^b^ According to the DSMB, the responsibility of lyophilized plasma could not be formally eliminated for patient with an embolic stroke who had received 150 mL of plasma. In this case, the causal hypotheses were the carotid wound, carotid clamping, carotid surgery, arterial hypotension and hypovolemia, and emergency anesthesia. The DSMB determined that this serious adverse reaction should not modify the course of the study.

^c^ Transfusion reactions were classified as febrile, allergic, or hypotensive reaction.

## Discussion

To the best of our knowledge, this is the first randomized study of prehospital use of lyophilized plasma by prehospital physician–staffed teams to evaluate the plasma effect on INR in posttraumatic hemorrhagic shock and associated coagulopathy. Allocation to lyophilized plasma was not associated with reduced INR values, and the trial yielded no convincing evidence to support the assumption that prehospital lyophilized plasma is effective for TIC.^23^ These results were consistent across the explored subgroups.

Examining these findings, we should consider the particularities of the emergency medical response system we studied. France’s established prehospital physician–staffed system and its effective response to a mass-casualty terrorist attack a few months before the study started are indicative of a mature emergency medical response system. On the other hand, a 2-tiered system has a cost in terms of ALS intervention time, and a longer delay in initiating plasma transfusion may have left little room for prehospital plasma to add value.^24^

We intended the present study to be pragmatic, with inclusion criteria easily applicable by prehospital ALS teams.^25^ Nevertheless, we had to adapt the design for highly constrained out-of-hospital settings. We broadened the inclusion criteria during data collection, but this decision may have contributed to the inclusion of patients with milder coagulopathy and yielded a less favorable benefit-risk balance for plasma transfusion.^26,27,28,29^

Our study confirms the feasibility and safety of prehospital plasma transfusion in its lyophilized form when provided by emergency medical response teams.^7,8^ Its use did not adversely affect the length of care or other treatment modalities.

Compared with previous studies, the PREHO-PLYO study did not reveal any change in INR at hospital arrival, whereas the PAMPer study^8^ showed an improvement and the COMBAT study^7^ showed a slight worsening of INR values in the plasma group. The rate of blunt trauma in the PREHO-PLYO study was similar to that in the COMBAT study and half that of the PAMPer study. This fact may have led to fewer patients with coagulopathy and may explain, at least in part, the lack of a visible plasma effect in our cohort. On the other hand, a post hoc analysis of the PAMPer and COMBAT trials showed the 20th minute of transfer was the inflection point for potential improvement in mortality. The PREHO-PLYO trial with a minimum transfer time of 30 minutes did not replicate this threshold.

Unlike in the PAMPer^8^ and COMBAT^7^ studies, most patients in the PREHO-PLYO study received tranexamic acid from the prehospital phase, which may have helped maintain fibrinogen levels within reference limits.^30^ The amount of crystalloid administered in the PREHO-PLYO plasma group was close to that administered in the PAMPer study and significantly higher than that in the COMBAT study. Patients received neither plasma first nor plasma only, which may also contribute to the nonsignificance of our results. Finally, this negativity supports the argument that mechanisms other than the one on which we focused underlie the benefits of prehospital plasma. Several proteins have been identified as markers of the plasma effect on the endothelial function and the inflammatory response.^4,31^ In addition, a secondary analysis of the PAMPer study^32^ showed that reduction of lactate levels mediated one-third of the plasma effect. In our study, which we did not design to replicate this finding, blood lactate levels in the hospital were similar in both groups (Table 1).

### Strengths and Limitations

This trial has several strengths. We consistently maintained group randomization until we evaluated all outcomes. No violation occurred regarding therapy allocation. The prehospital team aimed to achieve hemodynamic goals following guidelines for posttraumatic hemorrhagic shock (eFigure 1 in Supplement 1), which standardized the trauma resuscitation protocol for the control and plasma cohorts.

The trial also has some limitations, including those inherent to the open-label design due to the technical impossibility of blinding, but we minimized this issue with the statisticians’ blinding. The point-of-care device for prehospital INR measurement did not meet the field conditions. Consequently, we modified the primary outcome to enroll more patients. We did not adjust the power analysis after extending the inclusion criteria. This decision may have led to the recruitment of patients with less coagulopathy and increased the probability of a type II error. Nevertheless, the Injury Severity Scores were close to those found in the COMBAT and PAMPer studies.^7,8^ Other limitations include the use of a single parameter (INR value) to estimate TIC and the small sample size that limited survival and subgroup analysis. The base-excess missing values were not random and could not be imputed. Furthermore, the high rate of vasopressor use and the low rate of massive transfusion limit the comparability of our results with other studies and deserve further analysis.

## Conclusions

Our findings suggest that the transfusion of lyophilized plasma is feasible and safe in an emergency medical response setting among severely injured patients who are at risk for hemorrhagic shock. We found no significant difference between the effects of plasma transfusion and standard care with normal saline infusion on TIC in this patient population.

## Notes

Supplement 1. **eTable 1.** Enrollment by Site and Base **eTable 2.** Missing Data and Multiple Imputation Procedures **eTable 3.** Characteristics and Outcomes of Patients, Before and After the Change in Inclusion Criteria **eTable 4.** Primary Outcome in Complete Case Analysis After Multiple Imputation Procedure **eTable 5.** Characteristics of Patients According to Whether or Not They Received Both International Normalized Ratio (INR) Measurements by Point of Care **eTable 6.** Multinomial Logistic Regression Models Accounting for the Association Between the International Normalized Ratio and the Ratio of Plasma-to-Crystalloid Volume **eTable 7.** Main Surgical Procedures by Group in the First 24 Hours in the Modified Intention-to-Treat Population **eTable 8.** Protocol Violations by Study Group From April 1, 2016, to September 30, 2019 **eFigure 1.** Prehospital PREHO-PLYO Trial Intervention Schematic Operating Procedure **eFigure 2.** Clinical Trial Timeline With Important Dates and Patient Enrollment **eFigure 3.** Diagnostic Plots After Multiple Imputation for the Primary Outcome (INR at Hospital Arrival) and the Coagulation Factors **eFigure 4.** Observed Relationship Between INR Values and Ratio of Plasma-to-Crystalloid Volume Administered During the Prehospital Phase **eFigure 5.** International Normalized Ratio (INR) According to Subgroups **eFigure 6.** Prehospital Feasibility of French Lyophilized Plasma
eReferences

Supplement 2.
Trial Protocol

Supplement 3.
Nonauthor Collaborators

Supplement 4.
Data Sharing Statement
