# Supplementary material for: Prehospital Lyophilized Plasma Transfusion for Trauma-Induced Coagulopathy in Patients at Risk for Hemorrhagic Shock: A Randomized Clinical Trial
Source: JAMA Netw Open. 2022 Jul 26;5(7):e2223619. doi: 10.1001/jamanetworkopen.2022.23619 (PMC9327575; doi:10.1001/jamanetworkopen.2022.23619)
Supplement: Supplement 1. — eTable 1. Enrollment by Site and Base eTable 2. Missing Data and Multiple Imputation Procedures eTable 3. Characteristics and Outcomes of Patients, Before and After the Change in Inclusion Criteria eTable 4. Primary Outcome in Complete Case Analysis After Multiple Imputation Procedure eTable 5. Characteristics of Patients According to Whether or Not They Received Both International Normalized Ratio (INR) Measurements by Point of Care eTable 6. Multinomial Logistic Regression Models Accounting for the Association Between the International Normalized Ratio and the Ratio of Plasma-to-Crystalloid Volume eTable 7. Main Surgical Procedures by Group in the First 24 Hours in the Modified Intention-to-Treat Population eTable 8. Protocol Violations by Study Group From April 1, 2016, to September 30, 2019 eFigure 1. Prehospital PREHO-PLYO Trial Intervention Schematic Operating Procedure eFigure 2. Clinical Trial Timeline With Important Dates and Patient Enrollment eFigure 3. Diagnostic Plots After Multiple Imputation for the Primary Outcome (INR at Hospital Arrival) and the Coagulation Factors eFigure 4. Observed Relationship Between INR Values and Ratio of Plasma-to-Crystalloid Volume Administered During the Prehospital Phase eFigure 5. International Normalized Ratio (INR) According to Subgroups eFigure 6. Prehospital Feasibility of French Lyophilized Plasma eReferences [file jamanetwopen-e2223619-s001.pdf]

Supplementary Online Content

Jost D, Lemoine S, Lemoine F, et al; Prehospital Lyophilized Plasma (PREHO-PLYO) Study Group. Prehospital lyophilized plasma transfusion for trauma-induced coagulopathy in patients at risk for hemorrhagic shock: a randomized clinical trial. *JAMA Netw Open*. 2022;5(7):e2223619. doi:10.1001/jamanetworkopen.2022.23619

eTable 1. Enrollment by Site and Base
eTable 2. Missing Data and Multiple Imputation Procedures
eTable 3. Characteristics and Outcomes of Patients, Before and After the Change in Inclusion Criteria
eTable 4. Primary Outcome in Complete Case Analysis After Multiple Imputation Procedure
eTable 5. Characteristics of Patients According to Whether or Not They Received Both International Normalized Ratio (INR) Measurements by Point of Care
eTable 6. Multinomial Logistic Regression Models Accounting for the Association Between the International Normalized Ratio and the Ratio of Plasma-to-Crystalloid Volume
eTable 7. Main Surgical Procedures by Group in the First 24 Hours in the Modified Intention-to-Treat Population
eTable 8. Protocol Violations by Study Group From April 1, 2016, to September 30, 2019
eFigure 1. Prehospital PREHO-PLYO Trial Intervention Schematic Operating Procedure
eFigure 2. Clinical Trial Timeline With Important Dates and Patient Enrollment
eFigure 3. Diagnostic Plots After Multiple Imputation for the Primary Outcome (INR at Hospital Arrival) and the Coagulation Factors
eFigure 4. Observed Relationship Between INR Values and Ratio of Plasma-to-Crystalloid Volume Administered During the Prehospital Phase
eFigure 5. International Normalized Ratio (INR) According to Subgroups
eFigure 6. Prehospital Feasibility of French Lyophilized Plasma
eReferences

This supplementary material has been provided by the authors to give readers additional information about their work.

© 2022 Jost D et al. *JAMA Network Open*.

eTable 1. Enrollment by Site and Base

Advanced Life Support Base	Total Enrollment
Paris BSPP	86
Paris SMUR	
Lariboisiere	7
Beaujon	1
Mondor	1
Marseille BMPM	16
Marseille SMUR	8
Lyon	1
Annecy	9
Pau	2
Brest	3
Total	134

SMUR, Service Mobile d'Urgence et de Réanimation ; Brigade de sapeurs-pompiers de Paris ; BMPM, Bataillon de marins-pompiers de Marseille.

© 2022 Jost D et al. *JAMA Network Open*.

eTable 2. Missing Data and Multiple Imputation Procedures

Multiple imputation procedures were performed on the data of patients involved in the modified intention-to-treat analysis. The missing values ranged from 0% for some demographic variables to as high as 36·8% for data on coagulation factor V at hospital admission in the plasma group. On arrival at the hospital, the International normalized ratio value was not available for 6 (4·5%) patients because of technical and logistical problems. We addressed the issue of missing data using the state-of-the-art multiple imputation techniques including all analysis variables, under the assumption that missing values were missing at random.[1]

Multiple imputation was conducted using the chained equation. Logistic regression models were used to fill in missing values for binary variables, and predictive mean matching was applied to fill in missing values for continuous variables. Considering that the estimation of the fraction of missing information was 0·33, we used the 'mi impute chained' command in Stata 16, generating 50 imputed datasets, and visual inspection of imputation convergence led to the choice of 20 burn-in iterations.[2] Analyses run on each dataset were pooled according to Rubin's rules.[3] The multiple imputation diagnostic plots are represented in the figures below. We compared the distributions of the observed, imputed, and completed values, and the plots show the observed and completed values. We used the kernel- Epanechnikov density estimator for continuous variables, and we used a histogram for categorical variables. We used Kolmogorov- Smirnov statistics to compare the observed and imputed distributions of each continuous variable in imputed variables. The null hypothesis (i.e., observed and imputed data follow the same distribution) was rejected at P<0·05. The imputed results are presented as the main results because they are similar to the complete case analysis estimates.

Number and percentage of missing data for each imputed variable

Imputed variables	Total (n=134)		Standard-Care Group (n= 66)		Plasma Group (n=68)	
	n	%	n	%	n	%
Time from injury to arrival at hospital	1	0.7	1	1.5	0	0
Tracheal intubation (time 0)	1	0.7	0	0	1	1.5
Heart rate (time 0)	2	1.5	1	1.5	1	1.5
Glasgow coma scale (time 0)	3	2.2	1	1.5	2	2.9
DBP (time 30)	3	2.2	1	1.5	2	2.9
Glascow coma scame (hospital admission)	3	2.2	1	1.5	2	2.9
Haemoglobin (hospital admission)	3	2.2	2	3	1	1.5
Heart rate (hospital admission)	3	2.2	2	3	1	1.5
SBP (time 30)	3	2.2	1	1.5	2	2.9
Duration of stay in the ICU	4	3	2	3	2	2.9
Platelets value (hospital admission)	4	3	3	4.5	1	1.5
PT % (hospital admission)	5	3.7	4	6.1	1	1.5
Haemoglobin value (time 0)	6	4.5	4	6.1	2	2.9
INR value (hospital admission)	6	4.5	4	6.1	2	2.9
DBP (hospital admission)	7	5.2	4	6.1	3	4.4
DBP (time 0)	7	5.2	4	6.1	3	4.4
SBP (hospital admission)	7	5.2	4	6.1	3	4.4
SBP (time 0)	7	5.2	4	6.1	3	4.4
SpO2 (time 0)	8	6	4	6.1	4	5.9
Fibrinogen (hospital admission)	11	8.2	7	10.6	4	5.9
SpO2 (time 30)	11	8.2	6	9.1	5	7.4
Duration of hospitalization	13	9.7	8	12.1	5	7.4
Injury Severity Score (hospital admission)	21	15.7	11	16.7	10	14.7
Coagulation factor X (hospital admission)	38	28.4	25	37.9	13	19.1
Coagulation factor II (hospital admission)	42	31.3	23	34.8	19	27.9
Coagulation factor V (hospital admission)	45	33.6	20	30.3	25	36.8
Coagulation factor VII (hospital admission)	46	34.3	22	33.3	24	35.3

© 2022 Jost D et al. *JAMA Network Open*.

The number of missing data did not differ between the two groups (p=0·99).
n , %, Number and percentage of missing data; time 0: Time of advanced life support team's arrival in the field; time 30, 30th minute after advanced life support team arrival in the field; DBP, Diastolic blood pressure; SBP, Systolic blood pressure; SpO2, O2-saturation; PT%, Prothrombin level; POC, Prehospital point-of-care; ALS, Advanced life support.

© 2022 Jost D et al. *JAMA Network Open*.

eTable 3. Characteristics and Outcomes of Patients, Before and After the Change in Inclusion Criteria[a]

	Period "Before change in inclusion criteria" (n=40)	Period "After change in inclusion criteria" (n=94)	P-value
**Shock Index lower limit for inclusion**	1.3	1.1	
**Median shock index (IQR)[c] in included patients**	1.51 (1.31–1.68)	1.35 (1.19–1.64)	0.035
**Demographics**			
Median age (IQR) — yr	37.6 (27.6–48.1)	33.8 (24.9–48.9)	0.39
Male sex — No. (%)	33 (82.5)	77 (81.9)	0.94
**Vital status at the point of injury**			
Glasgow Coma Scale score <8 — No. (%)[b]	3 (7.5)	18 (16.1)	0.18
Median heart rate (IQR) — beats/min	115 (103–132)	111 (102–126)	0.34
Median systolic blood pressure (IQR) — mm Hg	76 (65–91)	83 (70–95)	0.22
Median diastolic blood pressure (IQR) — mm Hg	50 (38–59)	52 (41–62)	0.64
**Outcomes**			
Median laboratory INR (IQR)	1.21 (1.12-1.40)	1.20 (1.10-1.47)	0.88
Laboratory INR in a given range — No. (%)			
<1.2	15 (37.5)	42 (44.7)	0.36
1.2 to 1.5	17 (42.5)	28 (29.8)	
>1.5	8 (20.0)	24 (25.5)	
Massive transfusion within first 24 hours — No (%)	4 (10)	10 (10.6)	0.91
30-day survival — No (%)	6 (15)	19 (20.2)	0.48

Abbreviations: IQR, interquartile ranges; No., number; INR, International Normalized Ratio; ALS, advanced life support.

a Continuous variables were compared with the use of the Mann–Whitney U test, and categorical variables were compared using the Fisher's exact test. "Patients" refers to those included in the modified intention-to-treat analysis.

b Shock index was defined as heart rate divided by systolic blood pressure.

c Scores range from 3 to 15, with lower score indicating a reduced level of consciousness.

© 2022 Jost D et al. *JAMA Network Open*.

eTable 4. Primary Outcome in Complete Case Analysis After Multiple Imputation Procedure

		Complete case analysis		imputed model analysis	
		INR value, median(IQR)	n	INR value, median(IQR)	n
**Modified Intention-to-treat analysis**	**Plasma group**	1.20 (1.10-1.49)	66	1.21 (1.12-1.49)	68
	**Standard-care group**	1.20 (1.10-1.39)	62	1.20 (1.10-1.39)	66
**Per-protocol analysis**	**Plasma group**	1.20 (1.10-1.49)	64	1.20 (1.10-1.50)	66
	**Standard-care group**	1.21 (1.10-1.39)	60	1.21 (1.11- 1.39)	64

The modified intention-to-treat analysis included 134 subjects correspondant to all patients who underwent randomisation, excluding those who were deemed ineligible after randomisation . The per-protocol analysis included 130 patients with a confirmed diagnosis of hemorrhagic shock on hospital arrival. INR, International Normalized Ratio; IQR, Interquartile range.

© 2022 Jost D et al. *JAMA Network Open*.

eTable 5. Characteristics of Patients According to Whether or Not They Received Both International Normalized Ratio (INR) Measurements by Point of Care

	Patients for whom delta-INR value was available	Patients for whom delta-INR value was NOT available	P value
	n= 68	n=66	
**Study group**			
Standard care	35 (51.5%)	31 (47.0%)	0.60
Plasma	33 (48.5%)	35 (53.0%)	
**Demographics**			
Age (years)	34.1 (25.0 - 49.0)	34.1 (26.3 - 48.1)	0.91
Women	13 (19.1%)	11 (16.7%)	0.71
Men	55 (80.9%)	55 (83.3%)	
**Type of injury**			
Blunt	43 (63.2%)	37 (56.1%)	0.40
Penetrating	25(36.8%)	29 (43.9%)	
**Mechanism of injury**			0.72
Motor vehicle crash	24 (35.3%)	22 (33.3%)	
Stab wound	18 (26.5%)	22 (33.3%)	
Firearme	2 (2.9%)	4 (6.1%)	
Fall	19 (27.9%)	15 (22.7%)	
Other	5 (7.4%)	3 (4.6%)	
**Organ injured**			
Head	12 (17.6%)	12 (18.2%)	0.94
Thorax	17 (25.0%)	19 (28.8%)	0.62
Abdomen	15 (22.1%)	18 (27.3%)	0.48
Pelvis	11 (16.2%)	10 (15.2%)	0.87
Lower limb	16 (23.5%)	18 (27.3%)	0.62
Upper limb	10 (14.7%)	9 (13.6%)	0.86
**Time from first call to hospital admission**	91 (72 - 109)	87 (70 - 108)	0.40
**Prehospital scores at time 0**			
GCS < 8	20 (29.9%)	17 (26.6%)	0.68
Shock Index	1.32 (1.16 - 1.56)	1.48 (1.29 - 1.71)	0.017
**Prehospital treatment**			
Intubation	21 (30.9%)	17 (25.8%)	0.32
Norepinephrine	29 (42.7%)	39 (59.1%)	0.06
Tranexamic Acid	60 (88.2%)	57 (86.4%)	0.74
Crystalloids (ml)	750 (500 - 1000)	890 (625 - 1250)	0.09
**In-hospital massive transfusion**			
No	46 (67.7%)	50 (75.8%)	
Yes	22 (32.3%)	16 (24.2%)	0.30

Patient characteristics were similar between patients who received delta-INR measurement and those who did not, except for the shock index at time 0. Data are presented as median (IQR) and n (%).

© 2022 Jost D et al. *JAMA Network Open*.

eTable 6. Multinomial Logistic Regression Models Accounting for the Association Between the International Normalized Ratio and the Ratio of Plasma-to-Crystalloid Volume

A. Univariate Analysis

	INR < 1.2 (base outcome)	INR between 1.2 and 1.5		INR > 1.5	
	OR (95% CI)	OR (95% CI)	P value	OR (95% CI)	P value
Ratio of Plasma-to-Crystalloid Volume	1	0.69 (0.44-1.10)	0.17	0.72 (0.54-0.96)	0.026
Age, per year	1	0.99 (0.96-1.02)	0.48	0.98 (0.94-1.03)	0.46
ISS <16	1				
16 to 24		0.10 (0.01-7.2)	0.29	0.88 (0.09-8.59)	0.92
25 to 34		1.19 (0.20-7.09)	0.85	1.27 (0.16-10.20)	0.82
> 34		2.31 (0.52-10.32)	0.27	3?41 (0.66-17.75)	0.14

B. Multivariate Analysis

	INR < 1.2 (base outcome)	INR between 1.2 and 1.5		INR > 1.5	
	OR (95% CI)	OR (95% CI)	P value	OR (95% CI)	P value
Ratio of Plasma-to-Crystalloid Volume	1	0.66 (0.42-1.06)	0.081	0.71 (0.52-0.97)	0.030
Age, per year	1	0.98 (0.94-1.02)		0.98 (0.93-1.02)	0.34
ISS <16	1				
16 to 24		0.01 (0.01-72)	0.27	0.60 (0.06-6.84)	0.68
25 to 34		0.61 (0.09-4.19)	0.61	0.67 (0.07-6.21)	0.73
> 34		1.39 (0.26-7.49)	0.70	2.16 (0.36-12.9)	0.40

Unadjusted and adjusted analysis using a multinomial logistic regression model. The Independent variable was the INR at hospital arrival, with INR < 1·2 being the reference category. Primary independent variable was the ratio of plasma-to-crystalloid volume. Age and Injury Severity Score were included in the multivariable model irrespective of their significance in the unadjusted analysis. The analyses were computed on the patients of interest for the ratio calculation, i.e. the patients in the plasma group (n=68).

CI, Confidence interval; ISS, Injury Severity Score; INR, International normalized ratio; OR: Odds ratio.

© 2022 Jost D et al. *JAMA Network Open*.

eTable 7. Main Surgical Procedures by Group in the First 24 Hours in the Modified Intention-to-Treat Population

	Standard-Care Group n = 66[a]	Plasma group n = 68[b]	Total n = 134
Vascular /Embolization	12	8	20
REBOA[c]	1	0	1
Wound suture	7	11	18
Limb amputation	2	1	3
Chest drainage/ Thoracotomy	7	12	19
Cardiac surgery	2	1	3
Crane surgery	3	2	5
Laparotomy	6	10	16
Orthopedic	11	7	18
Urologic surgery	1	0	1

A patient could require multiple urgent surgical interventions within the first 24 hours.

[a] Data were unavailable for 17 in the control group and

[b] Data were unavailable for 12 in the plasma group.

[c] REBOA, Resuscitative Endovascular Balloon Occlusion of the Aorta.

© 2022 Jost D et al. *JAMA Network Open*.

eTable 8. Protocol Violations by Study Group From April 1, 2016, to September 30, 2019

	Normal saline Group n = 74	Plasma Group n = 76
No. of protocol violations	7	5
Enrolled without meeting the inclusion criteria		
- Age < 18 years	1	0
- Traumatic cardiac arrest	1	2
- Upper gastrointestinal bleeding	1	1
- Shock Index under the limit of study criteria	4	0
- plasma initiation only after hospital admission	0	1
Incomplete or lack of subject written consent	0	0
Breach of confidentiality	0	0
Incorrect version of informed consent signed	0	0
Other		
- Post Partum Hemorrhage	0	1

Protocol violations were sought in the intention-to-treat population (n=150).

© 2022 Jost D et al. *JAMA Network Open*.

eFigure 1. Prehospital PREHO-PLYO Trial Intervention Schematic Operating Procedure

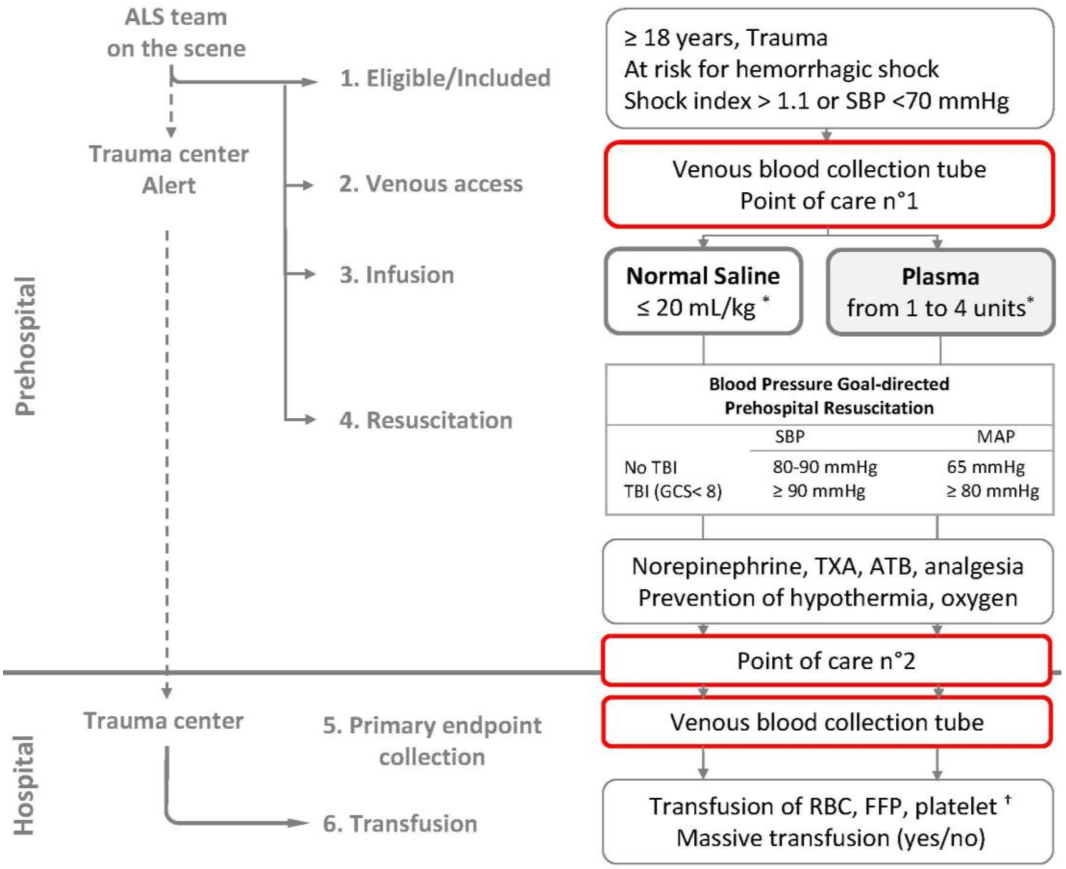

SBP, Systolic blood pressure; MAP, Mean arterial pressure; INR, International normalized ratio; RBC, Red blood cells; FFP, Fresh frozen plasma; GCS, Glasgow coma scale; TBI, Traumatic brain injury; TXA, Tranexamic acid; ATB, Antibiotic; O2, Oxygen; ALS, Advanced life support.

* Association with RBC transfusion if available.

† Transfusion continuation of plasma-units initiated in the prehospital setting.

© 2022 Jost D et al. *JAMA Network Open*.

eFigure 2. Clinical Trial Timeline With Important Dates and Patient Enrollment

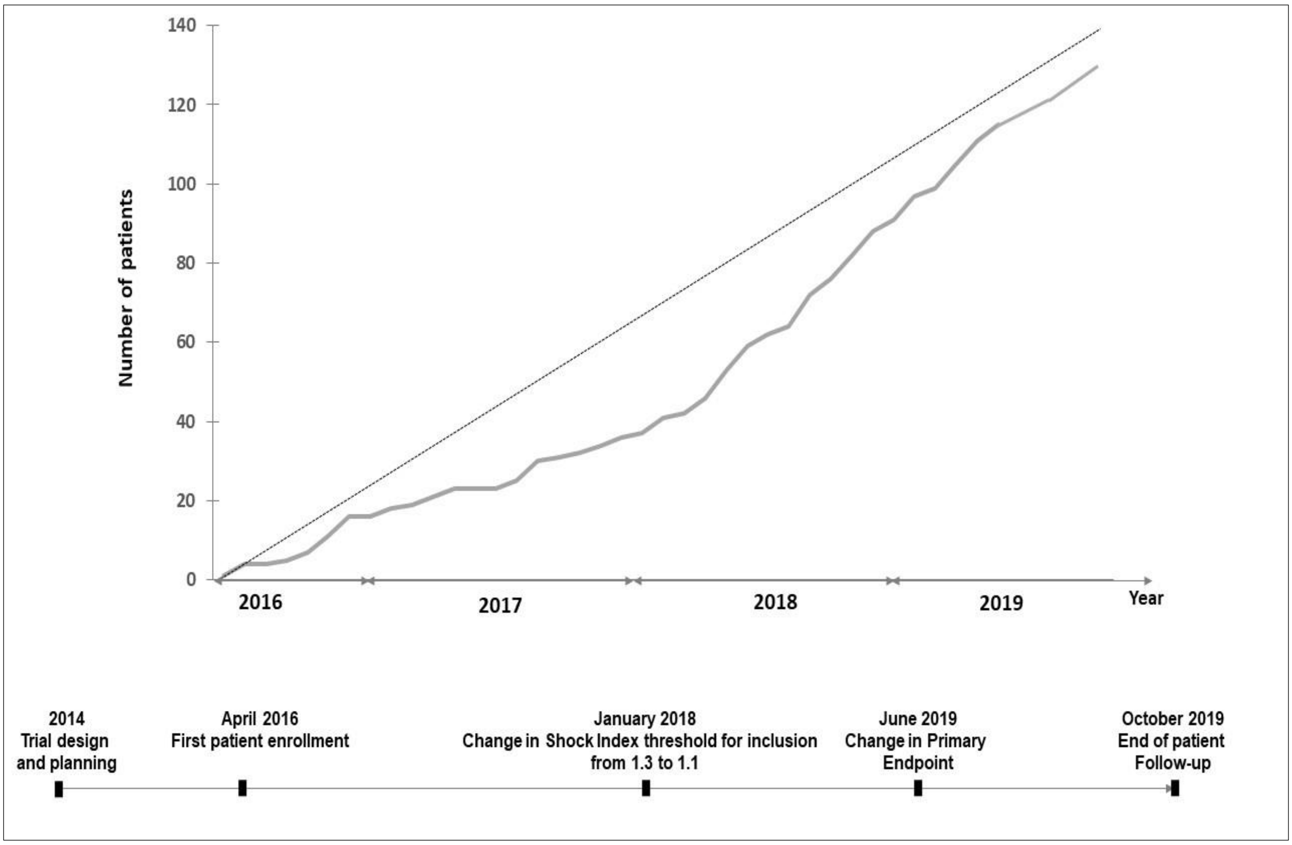

The dotted line indicates the expected inclusion rate. The solid line indicates the actual inclusion rate for patients included in the modified intention-to-treat analysis (n=134).

© 2022 Jost D et al. *JAMA Network Open*.

eFigure 3. Diagnostic Plots After Multiple Imputation for the Primary Outcome (INR at Hospital Arrival) and the Coagulation Factors

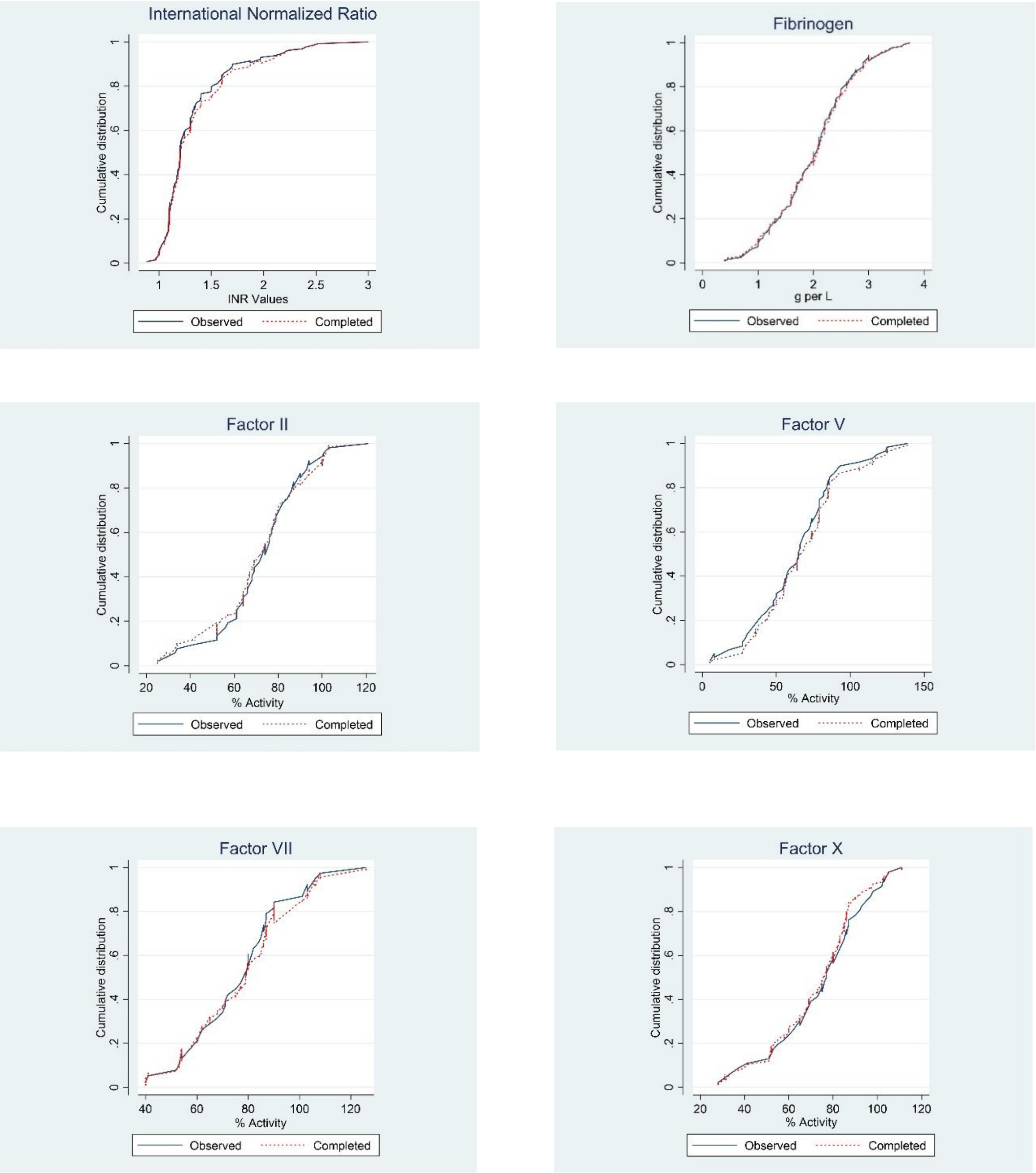

The red dotted lines and black solid lines show the distributions of the values of the variables before and after multiple imputation, respectively. The x-axis reports the range of values of the variable, the y-axis the cumulative distribution.

© 2022 Jost D et al. *JAMA Network Open*.

eFigure 4. Observed Relationship Between INR Values and Ratio of Plasma-to-Crystalloid Volume Administered During the Prehospital Phase

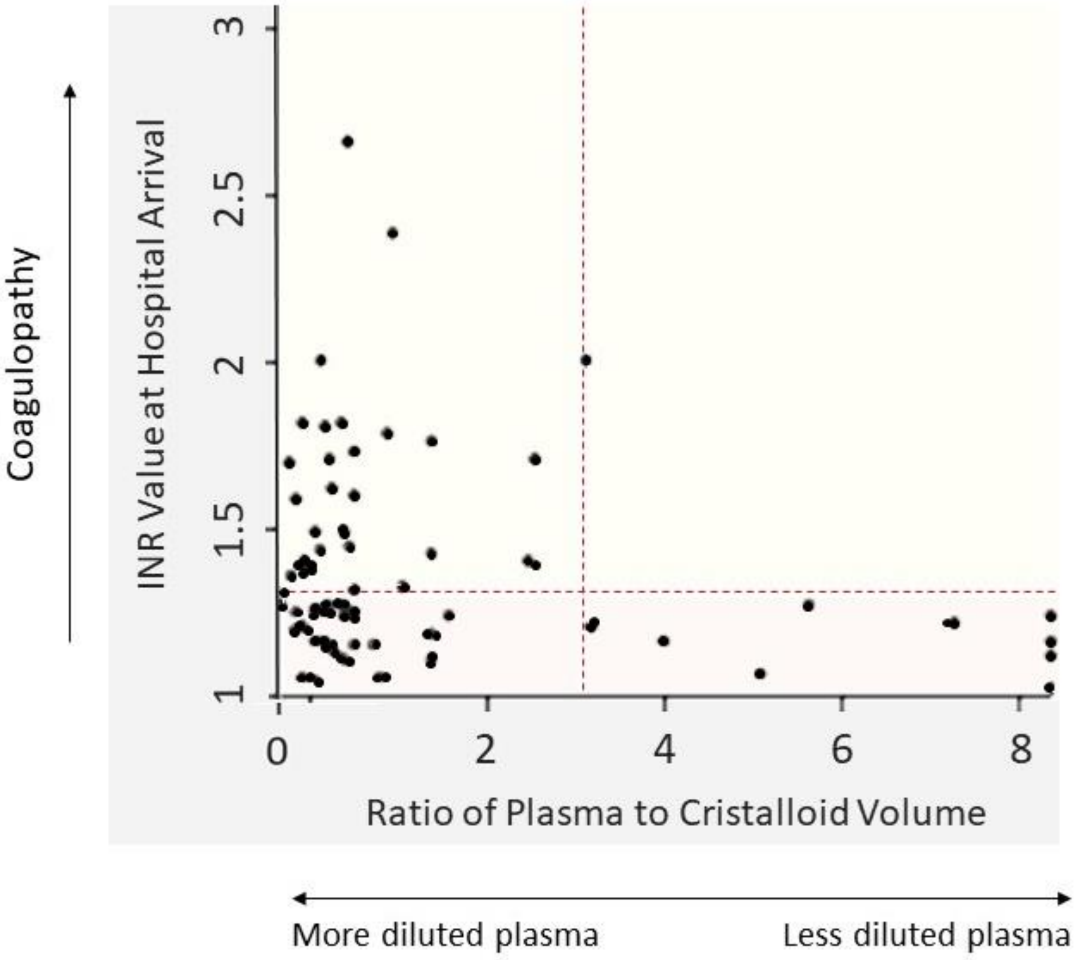

Two-way scatterplot of INR values versus the ratio of plasma-to-crystalloid volume for patients in the lyophilized plasma group (n=68). Higher INR values tend to be indicative of lower ratio of plasma-to-crystalloid volume values. Conversely, higher ratio of plasma-to-crystalloid volume tend to correspond to lower INR values. INR, international normalised ratio.

© 2022 Jost D et al. *JAMA Network Open*.

eFigure 5. International Normalized Ratio (INR) According to Subgroups

		No. of Patients	Control No. events/ Total	Plasma No. Events / Total	OR (95% CI)	P value
Penetrating trauma	No	80	27/40	25/40	0.8 (0.29 - 2.22)	0.64
p=0.87	Yes	54	14/26	12/28	0.64 (0.19 - 2.14)	0.42
Pelvis Injury	No	113	34/55	28/58	0.58 (0.25 - 1.30)	0.15
p=0.43	Yes	21	7/11	9/10	1.41 (0.32 - 6.37)	0.60
Prehospital use of vasopressors	No	66	17/31	18/35	0.87 (0.30 - 2.56)	0.78
p=0.52	Yes	68	24/35	19/33	0.62 (0.20 - 1.88)	0.35
Prehospital Intubation	No	96	27/50	23/46	0.85 (0.35 - 2.05)	0.69
p=0.73	Yes	38	14/16	14/22	0.25 (0.13 - 3.05)	0.39
Brain Injury Ctscan +	No	118	35/59	32/59	0.81 (0.37 - 1.80)	0.58
p=0.89	Yes	16	6/7	5/9	0.21 (0.003 - 3.40)	0.23
Injury Severity Score > 24	No	63	15/32	14/31	0.93 (0.31 - 2.82)	0.89
p=0.91	Yes	71	26/34	23/37	0.51 (0.15 - 1.59)	0.19
Massive transfusion (> 10 unit / 24 hrs)	No	100	27/51	21/49	0.67 (0.28 - 1.58)	0.31
p=0.41	Yes	34	14/15	16/19	0.38 (0.007 - 5.53)	0.61
Death up to 24 hrs	No	118	32/59	28/59	0.76 (0.36 - 1.73)	0.52
(no.total - no. at risk at 24 hrs) p=0.83	Yes	16	6/7	6/9	0.33 (0.005 - 6.01)	0.58
Total			41/66	37/68	0.72 (0.34-1.53)	0.37

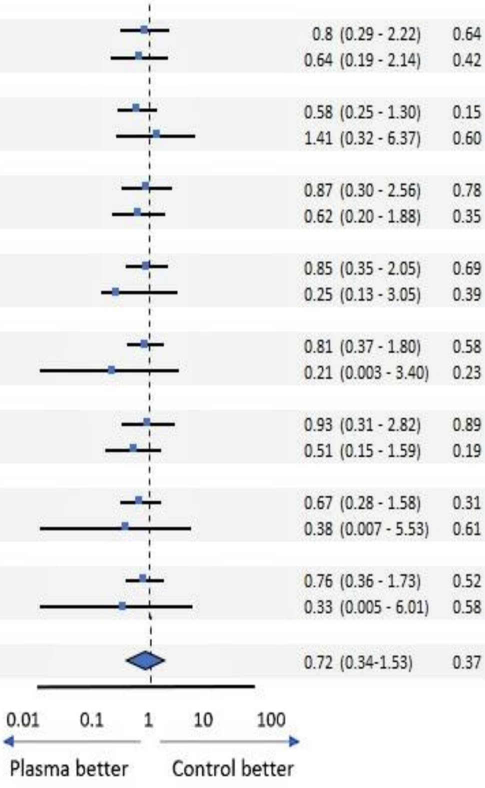

Odds ratios with 95% confidence intervals relative to an INR cutoff value of 1.2 in the different subgroups. The dotted vertical line indicates an Odds ratio of 1.0, i.e., no difference in treatment effect between the plasma and standard-care groups. The odds ratio scale is logarithmic. We detected no heterogeneity in the effect of trial-group assignment on INR value in any subgroup: p-value of the test of heterogeneity is mentioned under the name of each corresponding variable. CT scan, Computed tomography scanner.

© 2022 Jost D et al. *JAMA Network Open*.

eFigure 6. Prehospital Feasibility of French Lyophilized Plasma

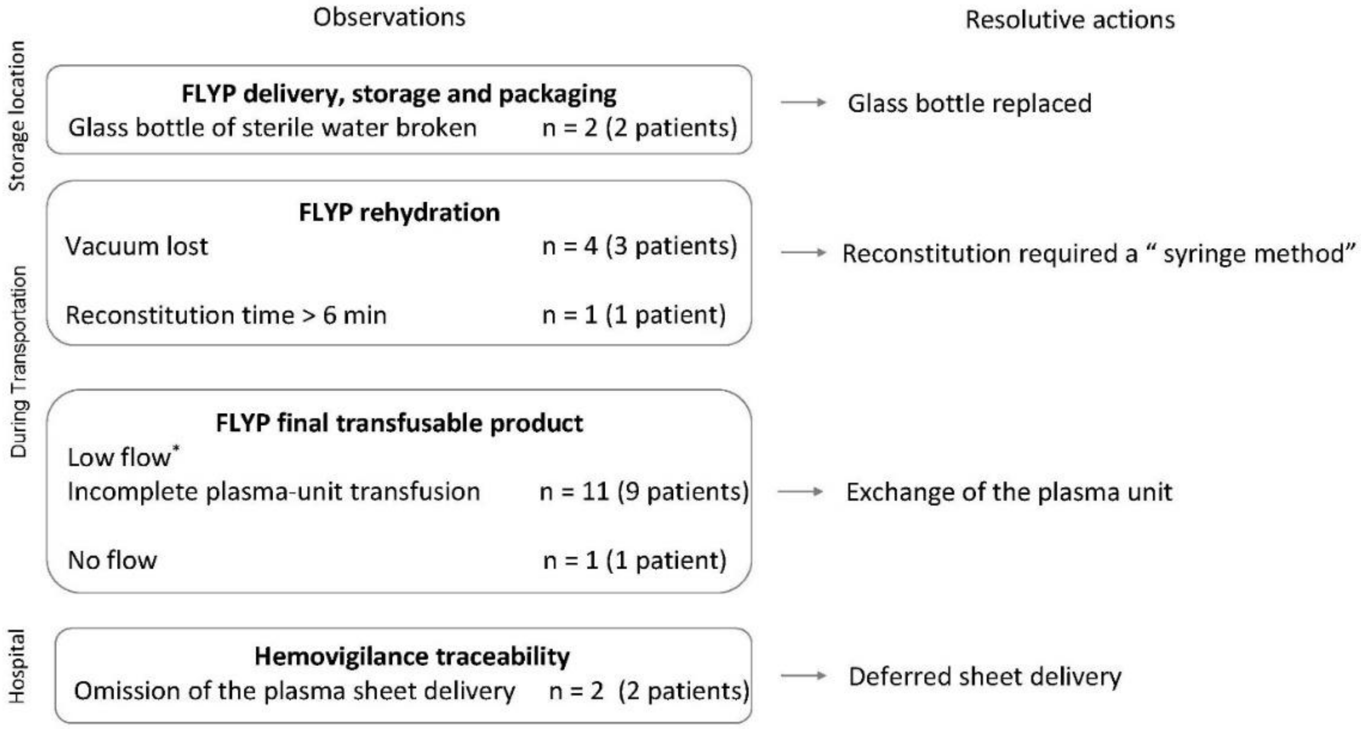

Logistical issues encountered from the delivery of the plasma by the French Military Blood Institute to the patient's arrival at the trauma center.

* Declarative data coming from the physician.

n, Number of bottles; min, Minute; FLYP, French lyophilized plasma.

© 2022 Jost D et al. *JAMA Network Open*.

eReferences

1 Schafer JL, Graham JW. Missing data: our view of the state of the art. *Psychol Methods* 2002; **7**: 147–77.

2 Austin PC, White IR, Lee DS, van Buuren S. Missing Data in Clinical Research: A Tutorial on Multiple Imputation. *Can J Cardiol* 2020; : S0828282X20311119.

3 Rubin DB, editor. Multiple Imputation for Nonresponse in Surveys. Hoboken, NJ, USA: John Wiley & Sons, Inc., 1987 DOI:10.1002/9780470316696.

© 2022 Jost D et al. *JAMA Network Open*.
