# Supplementary material for: Prehospital Lyophilized Plasma Transfusion for Trauma-Induced Coagulopathy in Patients at Risk for Hemorrhagic Shock: A Randomized Clinical Trial
Source: JAMA Netw Open. 2022 Jul 26;5(7):e2223619. doi: 10.1001/jamanetworkopen.2022.23619 (PMC9327575; doi:10.1001/jamanetworkopen.2022.23619)
Supplement: Supplement 2. — Trial Protocol [file jamanetwopen-e2223619-s002.pdf]

# Prehospital Lyophilized Plasma for Trauma-Induced Coagulopathy in Patients at Risk

# for Hemorrhagic Shock: A Multicentre Randomized Open-label Trial.

# The PREHO-PLYO Study.

ClinicalTrials.gov number: NCT02736812.

**Supplement 2.**

This appendix has been provided by the authors to give readers additional information about their work.

Corresponding Author: Dr Sabine Lemoine, Paris Fire Brigade Medical Emergency Department, 1 place Jules Renard, 75017 Paris, France.

sabine.lemoine@pompiersparis.fr, phone +33.01.56.79.67.53

This supplement 2 contains the following items:

Trial protocol (Version of May 27, 2019)
List of amendments
Statistical Analysis Plan (Version of May 27, 2019)

The PREHO PLYO Study

**Protocol version V6, May 27, 2019.**

PREHO PLYO Trial protocol - DCSSA: 2014RC04 BCR ID: 2015-A00866-43/6

*This document reports the final version of the trial protocol and includes all the modifications made during the study.*

The preho-plyo study group investigators and collaborators.

**Paris Fire Brigade Emergency Medicine Department :**

Michel Bignand, MD; René Bihannic, PharmD; Clément Derkenne MD; Pascal Dang Minh, MD; Maëlle Delacote, MD; Ludovic Delhaye, NP; Marilyn Franchin-Frattini, MD; Benoît Frattini, MD; Anne Godefroy, NP; Nicolas Hervault, NP; Vivien Hong Tuan Ha, MD; Daniel Jost,MD; Romain Jouffroy, MD; Isabelle Klein, MD; Vincent Lanoë, NP; Michael Lemaire, PharmD; Frédéric Lemoine, NP; Sabine Lemoine MD; Olga Maurin, MD; Nicolas Pouliquen, NP; Alexandre Petermann, MTA; Bertrand Prunet, MD; PhD Eloi Rouche, PharmD; Marina Salomé, CRA, MDM; Edouard Seguineau, MD; Olivier Stibbe, MD; Jean-Pierre Tourtier, MD, PhD; Julie Trichereau, Mstat; Stéphane Travers, MD,PhD.

**Firefighter Medical Emergency Department of Marseille :**

Sébastien Beaume, MD ; Franck Peduzzi, MD; François Topin, MD.

**Department of Education, Research and Innovation (DFRI), Service de Santé des Armées :**

Emilie Louis-Delauriere, CRA ; Christophe Renard, MD PhD ; Catherine Verret, MD.

**French army blood transfusion center (CTSA) :**

Eliane Garrabe, MD PhD ; Chantal Lutomski, HCE ; Christophe Martinaud, MD PhD; Anne C. Mendes, PharmD; Anne Sailliol, MD Ph.D

**French SAMU departments :**

Clémence Baudoin, MD **(**SAMU 75 Lariboisière Hospital)
Elise Brami, MD (SAMU 94 Henri Mondor Hospital)
Charlotte Chollet, MD (SAMU 94 Henri Mondor Hospital)
Maxime Diaz, MD (SAMU 92, Beaujon Hospital)
Pierre Y. Dubien, MDPhD (SAMU 69, Lyon Hospital)
François X. Duchateau, MD (SAMU 92, Beaujon Hospital)
Marc Fournier, MD (SAMU 13, Marseille Hospital)
Benjamin Garnier, MD (SAMU 29, Brest Hospital)
Pierre Y. Gueugniaud, MD PhD (SAMU 69, Lyon Hospital)
François Kerbaul, MD PhD (SAMU 13, Marseille Hospital)
Eric Lecarpentier, MD (SAMU 94 Henri Mondor Hospital)
Marc Leone, MD PhD (SAMU 13, Marseille Hospital)
Patrick Plaisance, MDPhD (SAMU 75 Lariboisière Hospital)
Marianne Sailliol, MD (SAMU 64, Pau Hospital).
Dominique Savary, MD (SAMU 74, Annecy Genevois Hospital)
Laurence Sénent, MD (SAMU 13, Marseille Hospital)
Benoit Vivien, MD PhD (SAMU 75 Necker enfants malades Hospital)

**French intensive care units participating teams :**

Sylvain Ausset, MD PhD (Percy Military Teaching Hospital)
Arie Attias, MD (Henri Mondor Hospital**)**
Deborah Benchetrit, MD (La Pitié Salpêtrière Hospital)
Salah Boussen, MD (Marseille Timone Hospital)
Marc Danguy-des-Déserts, MD (Clermont-Tonnerre Military Teaching Hospital)
Jean S. David, MD PhD (Lyon-south Hospital)
Jacques Duranteau, MD PhD (Bicêtre Hospital**)**
Etienne Escudier, MD (Annecy Genevois Hospital)
Emmanuelle Hammad, MD (Marseille Nord Hospital)
Didier Journois, MD PhD (European Georges Pompidou Hospital)
Olivier Langeron, MD PhD (La Pitié Salpêtrière Hospital)
Bernard Lenoir, MD PhD (Percy Military Teaching Hospital)
Albrice Levrat, MD (Annecy Genevois Hospital)
Claude-Denis Martin MD PhD (Marseille-North Hospital) (Marseille Timone Hospital)
Arthur Neuschwander, MD PhD (European Georges Pompidou Hospital)
Yves Ozier, MD PhD ((Brest Cavale Blanche Hospital)
Mehdi Ould-Ahmed, MD PhD (Begin Military Teaching Hospital)
Catherine Paugam, MDPhD (Beaujon Hospital)
Eric Peytel MD PhD (Marseille Laveran Military Teaching Hospital)
Mathieu Raux, MDPhD (La Pitié Salpêtrière Hospital)
Thomas Rimmele MD PhD (Lyon Edouard Herriot Hospital)
Karim Tazarourte, MD PhD (Lyon Edouard Herriot Hospital)
Jean P. Tourtier, MD PhD (Begin Military Teaching Hospital)

**Hospital Laboratories**

Michel Arock, PharMD PhD (La Pitié Salpêtrière Hospital)
Laurence Camoin-Jau, PharMD PhD (Timone Hospital)
Vincent Foissaud, MD (Percy Military Teaching Hospital)
Nathalie Ouéidat, PharmD (La Pitié Salpêtrière Hospital)
Emmanuelle de Raucourt, MD PhD (Beaujon Hospital)
David Smadja, PharmD PhD (European Georges Pompidou Hospital)
Philippe Vest, PharmD (Percy Military Teaching Hospital)

**And the French Blood Establishments of Annecy, Brest, Lyon, Marseilles, Paris, and Pau.**

Abbreviations :
CRA, Clinical Research Assistant
HCE, Health care executive
MDM, Medical Management
MStat, Master of Statistics
MTA, Medical Technical Assistant

**TABLE OF CONTENTS**


**TABLE OF CONTENTS** .......... **4**
**List of abbreviations** .......... **6**
**Summary** .......... **7**
**Exclusion criteria** .......... 8
**1.1 Description of the French Lyophilized Plasma (FLYP)** .......... 10
**1.2 Background** .......... 10
**1.3 Benefits and potential risks associated with the PREHO PLYO trial** .......... 11
**1.4 FLYP transfusion strategy** .......... 11
**1.5 Human subjects: Requirement for Emergency Research** .......... 12
**1.6 Study population.** .......... 12
**2 OBJECTIVES** .......... **12**
**2.1 Main objective** .......... 12
**2.2 Secondary objectives** .......... 12
**3 DESIGN** .......... **12**
**3.1 Outcome variables** .......... 12
**3.2 Methods** .......... 13
3.2.1 Study design .......... 13
3.2.2 Randomization .......... 13
**3.3 Study timelines** .......... 13
3.3.1 Prehospital phase .......... 13
3.3.2 Hospital phase .......... 14
3.3.3 Standard Operating Procedure for the biological tubes: .......... 14
3.3.4 Synopsis of the study .......... 15
3.3.5 Research timeline .......... 15
**3.4 Transfusion requirements and Transfusion stop** .......... 16
**3.5 Description and expected duration of an individual's participation in the research** .......... 16
**3.6 Definition and justification of the end of the research if it does not correspond to the end of the participation of the last person who takes part in the study.** .......... 16
**3.7 Description of the permanent or temporary stop rules** .......... 16
**3.8 Open-label** .......... 16
**3.9 Data to be collected directly in the CRF (source data)** .......... 16
**4 SCREENING and ENROLLMENT** .......... **17**
**4.1 Inclusion Criteria** .......... 17
**4.2 Non-inclusion Criteria** .......... 17
**4.3 Exclusion Criteria** .......... 18
**4.4 Procedure for transfusion reactions** .......... 18
**4.5 Hospital care follow-up if the research is stopped** .......... 18
**4.6 Arrangements for monitoring individuals involved in research based on, among other things, non-clinical data, including the duration of monitoring and follow-up procedures, and procedures for the medical management of individuals involved in research in the event of an emergency** .......... 18
**4.7 Procedures for obtaining consent from research subjects** .......... 18
**4.8 Concurrent participation in another research study and expected exclusion period at the end of the study** .......... 19
**5 TREATMENTS ADMINISTERED** .......... **19**
**5.1 FLYP** .......... 19
5.1.1 Indications .......... 19
5.1.2 Packaging of the product .......... 20
5.1.3 Conservation .......... 20
5.1.4 Labels .......... 20
5.1.5 Reconstitution .......... 20
5.1.6 Transfusion .......... 21
5.1.7 Disposition of units .......... 21
5.1.8 Plasma Traceability .......... 21
**5.2 PHYSIOLOGICAL SERUM** .......... 21
5.2.1 Indications .......... 21
5.2.2 Packaging and unit shape of the product .......... 21
5.2.3 Conservation .......... 21

5.2.4 Label ........ 21
5.2.5 Administration ........ 21
**5.3 Permitted and prohibited treatments in the protocol** ........ 21
**5.4 Monitoring of the infusion/ transfusion** ........ 22
**5.5 Practical procedures for accounting, storage, and delivery of experimental treatments within the investigating centers** ........ 22
**6 EVALUATION OF THE EFFECTIVENESS OF THE TREATMENT ........ 22**
**6.1 Description of effectiveness evaluation parameters.** ........ 22
6.1.1 Clinical parameters ........ 22
6.1.2 Biological parameters ........ 22
**6.2 Planned methods and timing for measuring, collecting, and analyzing effectiveness metrics** ........ 22
**7 SAFETY ASSESSMENT ........ 23**
**7.1 The vigilance during the trial** ........ 23
7.1.1 Definitions ........ 23
**7.2 Investigator's responsibilities** ........ 23
7.2.1 Notification of AE to the sponsor ........ 23
7.2.2 What should be declared? ........ 23
7.2.4 How to report? ........ 24
**7.3 Sponsor's responsibilities** ........ 25
**7.4 Planned methods and timing for measuring, collecting, and analyzing safety assessment metrics** ........ 25
**7.5 Data Safety Monitoring Board (DSMB/ CSI)** ........ 26
**7.6 Reporting of Adverse Events** ........ 26
**8 STATISTICS ........ 28**
**8.1 Analysis plan** ........ 28
**8.2 Anticipated number of people to be included in the research with its statistical justification and anticipated number of people in each research location** ........ 29
**8.3 Statistical significance** ........ 30
**8.4 Statistical analysis for stopping the research** ........ 30
**8.5 Missing or invalid data** ........ 30
**8.6 Substantive modifications to the research protocol** ........ 31
**8.7 Patients included in the analysis** ........ 31
**9 RIGHT OF ACCESS TO SOURCE DATA AND DOCUMENTS ........ 31**
**9.1 Privacy and Confidentiality** ........ 31
**10 QUALITY CONTROL AND QUALITY ASSURANCE ........ 32**
**11 ETHICAL CONSIDERATIONS ........ 32**
**12 DATA PROCESSING AND RETENTION OF DOCUMENTS AND RESEARCH DATA ........ 33**
**13 FINANCING AND INSURANCE ........ 33**
**14 References ........ 34**
**15 LIST OF AMENDMENTS ........ 36**
**16 STATISTICAL ANALYSIS PLAN. ........ 37**
**16.1 Background and rationale** ........ 38
**16.2 Objectives** ........ 38
**16.3 Trial design** ........ 38
**16.4 Randomization** ........ 38
**16.5 Sample size** ........ 39
**16.6 Trial Population** ........ 40
**16.7 Outcome definitions** ........ 41
**16.8 Missing data** ........ 42
**16.9 Additional analyses** ........ 43

## List of abbreviations

AE Adverse Event
ANSM Agence Nationale de Sécurité du Médicament et produit de Santé
AP-HM Assistance Publique- Marseille Hospital
AP-HP Assistance Publique - Paris Hospital
APTT Activated partial thromboplastin time
AR Ambulance de reanimation /Advanced life support team ambulance
ARDS Acute respiratory distress syndrome
BMPM Bataillon des marins-pompiers de Marseille
BP Blood pressure
BSPP Brigade de sapeurs-pompiers de Paris
CBC Complete blood count
CPP Comité de protection des personnes / Ethic Committee
CRA Clinical Research Associate
CRF Case Report Form
CTSA Centre de Transfusion Sanguine des Armées
EFS Etablissement français du sang / French Blood Agency
FFP Fresh frozen plasma
FFP-IA Fresh frozen plasma treated with amotosalen
FLYP Fresh lyophilized plasma
GCP Good Clinical Practice
GMP Good Manufacturing Practices
HR Heart rate
ICH International Conference Harmonisation
IGSII Indice de Gravité Simplifié II / Simplified severity index II
ISS Injury Severity Score
PPh Postpartum hemorrhage
PT Prothrombin levels
RBCs Red Blood Cells
SAE Serious Adverse Event
SAMU Service d'Aide Médicale d'Urgence / Prehospital emergency medical service
SMUR Service Mobile d’Urgence et de Réanimation / Prehospital emergency medical service
SpO2 Pulse Oxygen Saturation
TACO Transfusion-Associated Cardiac Overload
TRISS Trauma and Injury Severity Score
TTT Treatment
UMH Unité Mobile Hospitalière / Prehospital mobile intensive care unit

## Summary

| | |
|---|---|
| **Sponsor** | Central Direction of the Army Health Service (SSA) |
| **Coordinating Investigator** | Daniel Jost, MD<br>Paris Fire Brigade , Emergency Medicine Dept. |
| **Title** | Prehospital administration of lyophilized plasma to treat coagulopathy associated with post-traumatic hemorrhagic shock. The PREHO PLYO Trial |
| **ID RCB number** | 2015-A00866-43/6 |
| **Background and significance / Study rationale** | Acquired failures of hemostasis during severe bleeding promote and maintain bleeding. Lyophilized plasma (FLYP) has been administered many times in the military setting, but only non-randomized observational studies have been performed in this context. Furthermore, it has never been used in the civilian prehospital setting. Therefore, there are no prehospital studies available that have compared the currently recommended treatment of hemorrhagic shock without FLYP versus with FLYP. |
| **Hypothesis** | We hypothesize that in the civilian out-of-hospital setting, early FLYP administration prevents or corrects post-traumatic coagulopathy. |
| **Objectives** | ***Primary Objective***<br>To measure the efficacy of FLYP on post-traumatic coagulopathy in the prehospital setting.<br><br>***Secondary Objectives***<br>- Feasibility of FLYP use in the prehospital setting<br>- Measuring the effect of FLYP on fibrinogen levels<br>- Measuring the effect of FLYP on transfusion requirements after hospital admission<br>- Measuring the effect of FLYP on the length of stay in the intensive care unit<br>- Measuring the effect of FLYP on the 30-day survival rate |
| **Research Design** | Multicenter, randomized, open-label controlled pragmatic trial. From the prehospital phase, the FLYP group receives plasma transfusion, symptomatic and etiological treatments. The standard-care group receives normal saline as well as symptomatic and etiological treatments. |
| **Inclusion criteria** | Adult patients over 18 years<br>Severely injured (blunt or penetrating trauma)<br>At risk for hemorrhagic shock and associated coagulopathy.<br>Systolic blood pressure <70 mmHg or shock index value (heart rate/ systolic blood pressure) over 1.1, at any time of the prehospital care. |
| **Non-inclusion criteria** | - Refusal of the person to participate in the research<br>- No affiliation with a healthcare protection system<br>- Age < 18 years |

| | |
|---|---|
| | - A person deprived of liberty<br>- Person subject to a safeguard of justice measure<br>- Unrectified Legionnaire<br>- Pregnancy<br>- History of known allergies to amotosalen and psoralens<br>- Administration of coagulation factors other than FLYP<br>- Patient in cardiac arrest before the arrival of professional rescuers |
| **Exclusion criteria** | - Non-usable blood sample<br>- Refusal to participate by the patient |
| **Treatments** | - FLYP (up to a maximum of 4 x 200 ml bottles)<br>- Normal saline (no limit required) |
| **Endpoints** | **Primary Endpoint:**<br>- INR at hospital arrival<br><br>**Secondary Endpoints:**<br>- INR-value variation between prehospital and in-hospital setting<br>- Collection of technical and logistical difficulties encountered before, during, and after the administration of FLYP.<br>- Variation in fibrinogen values between prehospital and hospital settings.<br>- Numbers of RBCs, platelet units, fibrinogen doses, clotting factors, plasma units needed during the first 24 and 48 hours<br>- Total length of stay in intensive care<br>- Number of days of hospitalization.<br>- 30-day survival rate. |
| **Required Sample Size** | 140 patients (70 in each group) |
| **Expected number of prehospital study site investigators** | Eight investigating centers (BSPP, SAMU 75, SAMU 69, SAMU 74, BMPM, SAMU 13, SAMU 29, SAMU 64) |
| **Duration of the trial** | Estimated duration of the inclusion period: 30 months<br>Time of participation for each patient: 30 days |
| **Statistical analysis** | • Description of patients' characteristics in the two groups for all collected variables of interest.<br><br>Primary endpoint:<br>• Comparison of the median values of INR at hospital admission between the two groups<br><br>Secondary Endpoints:<br><br>• Comparison of the median values of fibrinogen at hospital admission<br>• Comparison of delta-INR by group: generalized estimating equation method<br>• Comparison of blood loss, volumes infused or transfused, the quantity of amines used between the two groups (Fisher exact, Student, Mann-Whitney tests).<br>• Comparison of the length of stay in intensive care between the two groups (Student, Mann-Whitney test)<br>• Survival analysis up to 30 days (Kaplan-Meier Curve, log-rank test) |

| | |
|---|---|
| | • A multiple-imputation process will manage missing variables<br>• Exploratory subgroup analysis<br>• Secondary exploratory analyses to further explain the primary outcome. |
| **Patient Benefit** | This study may change the recommendations for the initial management of patients in hemorrhagic shock in both civilian and military settings. |

**SCIENTIFIC JUSTIFICATION AND DESCRIPTION OF THE RESEARCH**

## 1.1 Description of the French Lyophilized Plasma (FLYP)

French lyophilized plasma - FLYP is currently distributed to military medical and surgical units deployed in overseas operations (OPEX) to meet the logistical constraints of the operational context. The need is to have therapeutic plasma available without delay to treat hemorrhagic casualties.
In the civilian setting, FLYP could be used by health care institutions for which there exist significant logistical difficulties that do not allow them to maintain a negative cold chain or in emergencies where therapeutic plasma is needed immediately. FLYP should be used until the fresh frozen plasma is thawed and available.

FLYP is manufactured from pooled fresh frozen plasma (FFP) obtained from less than 11 donors by apheresis (donors of blood groups A, B, and AB, free of hemolytic anti-A or anti-B immune antibodies, initially stored at -25°C or below).
The mixture, in chosen proportions, allows obtaining a plasma compatible with all blood groups (universal plasma).

FLYP is sterile and comes in the form of a powder with a residual moisture < 2%. It is packaged in a sterile, non-pyrogenic glass bottle.

## 1.2 Background

Acute hemorrhage is the leading cause of mortality in severe trauma, both in the field and among severely injured patients who arrive alive at the hospital [1,2]. Acquired hemostasis failures during severe bleeding promote and maintain bleeding. Coagulopathy is found in 30 to 50% of cases in severely injured patients, whether civil or war-related and is associated with a mortality rate estimated at approximately 40%. [3]
The mechanisms of coagulopathy are complex and result from the interaction between tissue damage, shock, or tissue hypoperfusion. [4] Acidosis, hypothermia, and hemodilution aggravate the prognosis.[5]

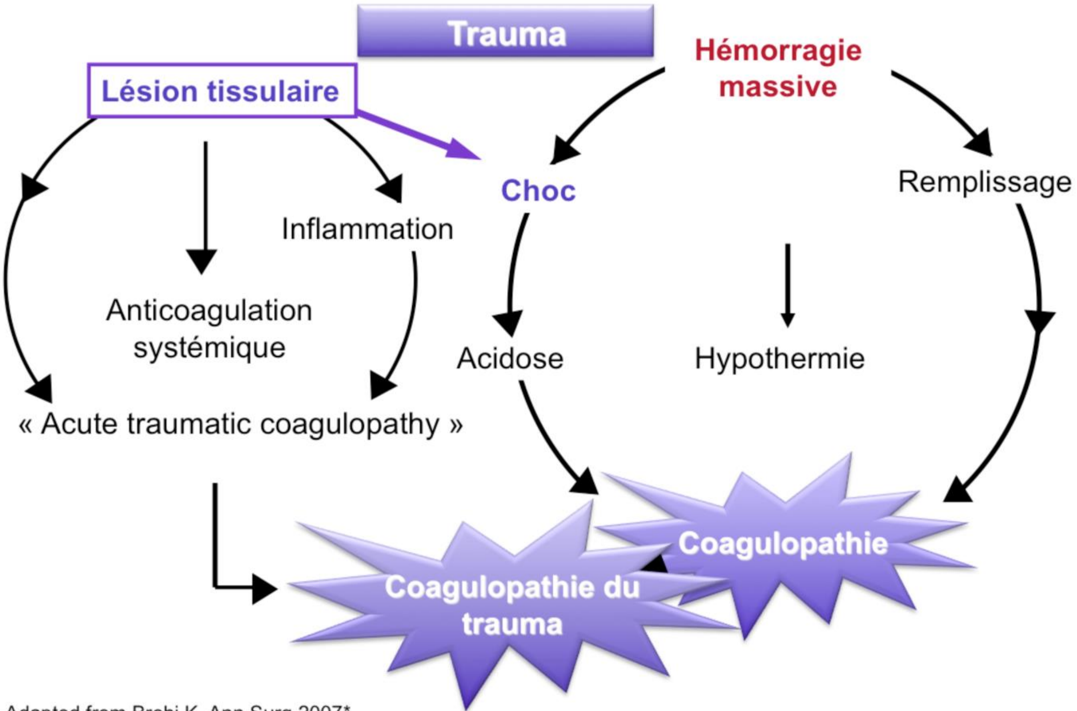


Adapted from Brohi K, Ann Surg 2007*

Improved knowledge of pathophysiological mechanisms has allowed earlier and more active management of the diagnostic and therapeutic management of this coagulopathy. Thus, in massive traumatic hemorrhage, there is a dramatic decrease of fibrinogen, platelets, and, more generally, coagulation factors.[6]

Rapid correction of hemostasis is essential, and plasma can prevent or correct this post-traumatic coagulopathy early by providing coagulation factors.

Mortality and morbidity are reduced when transfusion is initiated as early as possible and when a plasma: RBC ratio of 1:1 is used from the start.[7] The importance of early transfusion without delay has been highlighted by De Biasi [8]. In this cohort study of massively transfused trauma patients, the authors highlighted the association between the delay in initiating plasma transfusion at a high ratio and survival. It is important to note that delay in plasma transfusion during the first hour increases mortality.

Fresh Frozen Plasma (FFP) in the field is subject to a thawing phase (30 minutes) and a more or less long transport phase, making its use in the prehospital phase exceptional. However, the FLYP of the French Military Blood Institute (CTSA) has proven its effectiveness in managing massive hemorrhage[9–12]. Its storage at room temperature, easy reconstitution in less than 6 minutes, and compatibility with all blood types make it immediately and rapidly available in the prehospital setting.

The in-hospital reference treatment is Fresh frozen plasma (FFP). Unfortunately, this treatment is not available in the civilian out-of-hospital setting. There are, therefore, no prehospital studies available that have compared the currently recommended treatment for hemorrhagic shock without FLYP versus with FLYP. Therefore, our study proposes the addition of FLYP as an innovative strategy to combat the development of coagulopathy in hemorrhagic shock in the prehospital phase. [6,13–15]

### 1.3 Benefits and potential risks associated with the PREHO PLYO trial

Bleeding is the leading cause of mortality in severe trauma patients who arrive alive at the hospital. In addition, a specific coagulopathy is found in about 30% of cases in injured patients, civil or war, and is associated with a mortality estimated at 40%.
Recent studies have shown that aggressive and early management of this coagulopathy is associated with an improved vital prognosis.

The use of FLYP allows for the early provision of coagulation factors while respecting the balance between activators and inhibitors and without the risk of increasing coagulopathy by filling with colloids or crystalloids while waiting for the plasmas to thaw.

**Expected benefits for patients in this study**

The expected benefits for those participating in this study are improved management at a decisive early stage of post-traumatic coagulopathy.

**Benefits to society:**

This study should improve knowledge of the effects of early plasma transfusion on patients in hemorrhagic shock and expand the indications for FLYP by bringing this treatment to the civilian population with the specific indication of trauma-induced hemorrhage.

**Known risks and constraints**

There are no known risks specific to the use of FLYP. The effect of cryodessication on the concentration of coagulation factors is a parameter that is monitored during manufacturing. The observational study on the use of FLYP in OPEX and certain exceptional situations, conducted since 2010 by the CTSA, has proven its tolerance and effectiveness [10].

In sporadic cases, transfusion of FFP may result in allergic reactions or respiratory complications (TACO).
It is necessary to observe whether the same clinical events occur with FLYP, knowing that its manufacturing method reduces this risk, as pooling minimizes the risk of immunological intolerance.

### 1.4 FLYP transfusion strategy

Treatment of traumatic coagulopathy is based primarily on the provision of plasma coagulation factors and platelets.

FLYP has all the qualities of therapeutic plasma. It can be reconstituted in less than six minutes. It is universally used for ABO blood groups and can be stored at room temperature. Its sophisticated galenic form should be reserved for situations where significant logistical difficulties (impossible to ensure a negative cold chain or for extreme emergency situations where therapeutic plasma must be supplied without delay).

FLYP transfusion is recommended only in cases of severe coagulopathy with the collapse of all coagulation factors, acute bleeding with global coagulation factor deficiency, and complex coagulation factor deficiency when specific coagulation fractions are not available.

As a general rule, the transfusion should be performed rapidly (210 ml in 5 to 30 minutes) but should always be adapted according to the patient's clinical parameters. During prehospital management, a maximum of four units of FLYP will be administered per patient (dose recommended by the CTSA) depending on the patient's tolerance.
The speed of administration will be 5 to 15 minutes per unit in this context.

### 1.5 Human subjects: Requirement for Emergency Research

The research will be conducted following the trial protocol, the Good Clinical Practices (GCP), and the legislative and regulatory processes.

The CTSA's production of FLYP units complies with the rules established by the Good Transfusion Practices and the Good Manufacturing Practices applied to injectable products, Lifesaving Blood Products, and sterile products. It should be noted that the ANSM regularly inspects the CTSA in this context.

Each investigator agrees to provide free access to all source data required by the sponsor's monitor, persons conducting audits, and members of the CCP (Ethics Committee) or competent authorities.

The investigators, persons in charge of quality control, monitors, clinical research assistants, auditors and all persons called upon to collaborate in the trials, will respect legislative and regulatory recommendations, in particular, articles L. 1121-3 and R-5121-13 of the public health code, and will take all the necessary precautions to ensure the confidentiality of the information relating to the patient and the medicinal-trial products.

### 1.6 Study population.

The study population consisted of adult patients with a hemorrhagic shock of traumatic origin managed by a prehospital medical team.
FLYP has been used for many years in traumatic contexts in the military environment. The population studied is justified by its belonging to civilian society, whose epidemiological profile is different from the military environment and deserves to be explored.

## 2 OBJECTIVES

### 2.1 Main objective

To show the effectiveness of FLYP administered prehospital during the management of hemorrhagic shock of traumatic origin, on the occurrence or treatment of post-traumatic coagulopathy.

### 2.2 Secondary objectives

- Feasibility of FLYP administration in the prehospital setting
- Show that FLYP improves fibrinogen levels
- To show that the contribution of FLYP decreases the transfusion needs (RBC, plasma, coagulation factors, platelets)
- To show that the contribution of FLYP decreases the length of stay in intensive care
- Compare mortality between the two groups until Day-30

## 3 DESIGN

### 3.1 Outcome variables

**Primary endpoint:**

- The primary endpoint is the international normalized ratio (INR) at hospital arrival.

**Secondary endpoints:**

- The delta-INR value between the prehospital INR and the INR at hospital arrival. The Point of Care Device Coaguchek® will measure these two INR values
- Listing of technical and logistical difficulties encountered before, during, and after the administration of FLYP.
- The change in fibrinogen level between hospital admission and prehospital setting. The number of RBC units, platelet units, fibrinogen, plasma units, and quantity of coagulation factors administered during the first 24 and 48 hours.
- Length of stay in intensive care
- Length of hospitalization
- 30-days survival.

## 3.2 Methods

### 3.2.1 Study design

This trial is a multicenter, randomized, controlled, open-label pragmatic study.

### 3.2.2 Randomization

Randomization assigns either the PLYO treatment combined with the usual treatment or the usual treatment alone.

CTSA randomizes study treatments in blocks of 2 boxes (one with PLYO, one without).
The treatment boxes are numbered in ascending order from 1 to 140.

The CTSA and the EFS will distribute the numbered boxes to the prehospital investigating centers as they are included (at the request of the centers) and by ascending order from 1 to 140. Therefore, only one package will be available in each ALS vehicle at the time of patient management.

The randomization list will be maintained by CTSA and SSA.

## 3.3 Study timelines

The study comprises two phases of investigation: the prehospital phase and the hospital phase.

### 3.3.1 Prehospital phase

This phase is performed by a prehospital investigator after verification of inclusion and non-inclusion criteria.
During this phase, no fibrinogen or other coagulation factors, other than FLYP

Before the administration of the experimental treatment, the management of a patient will be :

- Stop the bleeding (tourniquet or hemostatic dressing or pressure point if necessary)
- Measure clinical and biological parameters (HR, BP, SpO2, hemoglobin level,...)
- Take a sample (1 drop of blood) to measure the prehospital PT/INR with the Coaguchek®.
- If possible, take a venous sample before any drug administration, using two blue tubes (PT, aPTT, fibrinogen, coagulation factors) and two purple tubes (blood group, platelets, etc.).

Particular attention must be applied to the filling of the tubes, which must be COMPLETE so that the results of the analyses can be interpreted.

- Ventilatory support with oxygen supply (high concentration mask or orotracheal intubation and mechanical ventilation)
- **<u>For the FLYP (+) group</u>: Administration of FLYP, up to 4 units (800ml)**

Plasma units are reconstituted as needed.
Any connected and opened bottles are left with the patient.

- **<u>For the FLYP (-) group:</u> Administration of saline solution,** as much as necessary to reach the goals recommended by the guidelines.

Then, for the two groups, according to the patient's clinical parameters:

- Use of amines, and tranexamic acid
- The dosage of crystalloids and the use of amines should make it possible to reach the goals recommended by the guidelines.

### 3.3.2 Hospital phase

**At hospital arrival:**

- Biological samples ( PT, aPTT., fibrinogen, CRP, and coagulation factors)
- Administration of the usual treatments.
- Follow-up by the hospital-based investigator for 30 days (
  - patient status (alive, deceased)
  - date of discharge from the intensive care unit and the hospital
  - follow-up of AEs, SAEs
  - Hemostasis test results collection.

All procedures performed during this phase will conform with the standard practices in use in each hospital.

Biological samples will not be stored after their usual processing.

### 3.3.3 Standard Operating Procedure for the biological tubes:

Prehospital blood samples are part of the usual diagnostic process of post-traumatic coagulopathy.

Prehospital blood samples will be collected prior to administration of the study treatment and dispatched to the hospital laboratory through each hospital's dedicated circuit.

The prehospital physician will write on the tube of collected blood sample :

- The patient's first and last name
- The patient's inclusion number
- The name of the prehospital center
- Date and time of sampling

A "patient label" (with name, first name, date of birth, date of hospital admission) will be created when the patient arrives in the hospital. This label will be fixed on the tube to facilitate the procedures of the laboratory technicians.

The prehospital physician will complete the blood test request sheet and will precise the indication "SAMPLE COLLECTED BEFORE ANY PLASMA TRANSFUSION."

**Samples collected after hospital admission** will follow the same process. But the blood sample analysis request sheet will be filled out by the Senior Physician in charge of the hospital admission unit. The request sheet will be precise with the indication: "POST FLYP TRANSFUSION."

The prehospital physician who transported the patient to the hospital makes the final check with the admission unit to ensure that the laboratory request forms have been completed correctly
and that the tubes have been correctly labeled and placed in the dedicated container (plastic bag).

Particular attention should be paid to identifying samples and the storage of samples corresponding to the coagulation factors. Care should be taken to ensure that the time for analysis and determination of coagulation factors is less than 6 hours. Samples should be stored at room temperature.

The biological samples directly related to the study will not be stored after their analysis.

### 3.3.4 Synopsis of the study

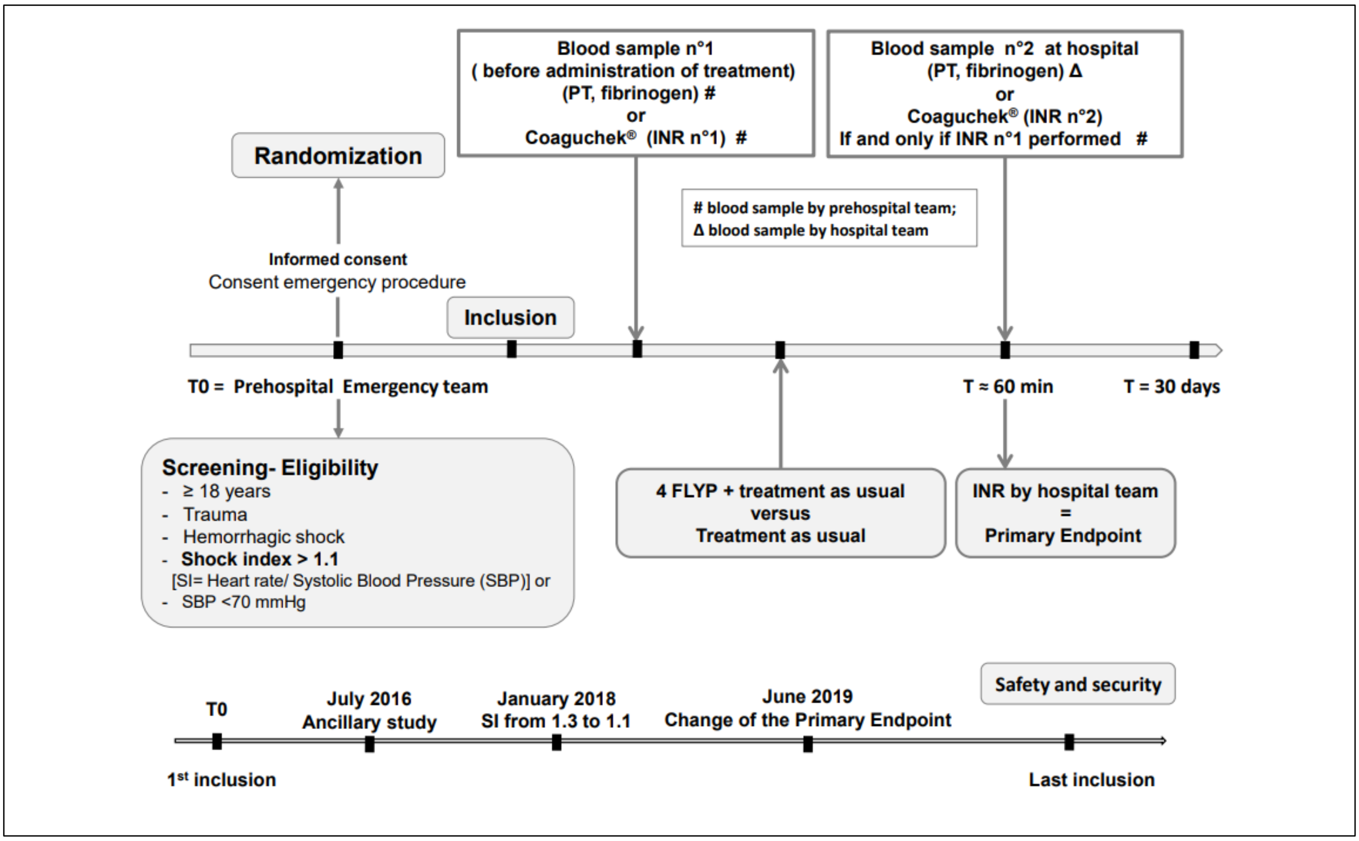


T, Time; PT, Prothrombin time level; INR, International normalized ratio; FLYP, French lyophilized plasma.

### 3.3.5 Research timeline

| | Phase Prehospital | Hospital phase | | |
|---|---|---|---|---|
| | | Admission | Follow-up | End of study |
| Consent or follow-on consent | X | X | X | X |
| Background | X | X | | |
| Verification of inclusion, non-inclusion and exclusion criteria | X | X | X | X |
| Clinical examination | X | X | X | X |
| Biology:<br>• PT/INR<br>• Prehospital INR<br>• TCA and fibrinogen<br>• platelets<br>• CRP | <br>X<br>X<br>X<br>X<br><br>X | <br>X<br><br>X<br>X<br>X<br>X | | |

| | | | | |
|---|---|---|---|---|
| • Blood Group | | | | |
| Randomization | Up to 4 FLYP + reference TREATMENT Or reference Treatment | | | |
| Adverse events | X | X | X | X |

### 3.4 Transfusion requirements and Transfusion stop

Maximum dose of experimental treatments administrated during the inclusion phase: 4 units of FLYP versus normal saline (enough to achieve recommended goals)
Criteria for stopping the plasma administration: in case of signs of allergia or clinical symptoms of intolerance to the treatment.
The patient will also be given normal saline as part of the usual management.

### 3.5 Description and expected duration of an individual's participation in the research

The total duration of participation for a patient in the study will be 30 days.

From the first inclusion, the sponsor must inform the competent authority and the CPP ((Ethics Committee) without delay of the effective start date of the study. In addition, the sponsor will transmit the end date of the study to the ANSM and the CPP (Ethic Committee) within 90 days.
The duration of the study is estimated at 30 months.

### 3.6 Definition and justification of the end of the research if it does not correspond to the end of the participation of the last person who takes part in the study.

<u>The end date of the research is the end of the participation of the last person involved in the study.</u>

The study will be prematurely stopped if unexpected serious adverse events occur. Similarly, unanticipated events or new information about the product may cause the sponsor to discontinue the study prematurely.

In case of premature termination of the study, the sponsor will transmit the information within 15 days to the ANSM and the CPP (Ethic Committee).

### 3.7 Description of the permanent or temporary stop rules

**- of an individual's participation in research:**

Each patient may withdraw from the study after the decision of the competent administrative authority, the sponsor, or the investigator, but also after the decision of a co-investigator or the patient himself in accordance with the regulations and as mentioned in the consent form.

In all cases, the investigator should document the reasons for the subject's discontinuation in the subject's medical record.

**- of part or all of the research:**
The study may be prematurely terminated in the event of unexpected serious adverse events (which may or may not require a review of the product's safety profile) or the occurrence of unanticipated events or new information about the product by the Independent Monitoring Committee (Data safety monitoring board).

### 3.8 Open-label

A blinded study cannot be performed because of the difficulties of manufacturing a placebo.
The study is therefore conducted in an open-label setting.

### 3.9 Data to be collected directly in the CRF (source data)

**Data collected in prehospital:**

Age,

gender

Circumstances

- ALS team delays (departure time, presentation time)
- Description of lesions, their type, and the organ injured

- Clinical parameters at ALS team arrival (HR, BP, RR, SpO2, Temperature, Hemoglobin) and their time of collection
- TREATMENT administered (FLYP or normal saline), and adverse reaction to administration (e.g., erythema), time of administration
- Filling performed: nature of products, quantity, time of administration
- Use of amines: name, dose (before/after FLYP or normal saline administration), time of administration
- Use of tranexamic acid or blood-derived medication (fibrinogen, prothrombin complex, etc.): dose, time of administration.
- Type of ventilatory support: High concentration mask, IOT-VM (FiO2 and ventilatory parameters noted), time of ventilatory management
- Chest tube +- autotransfusion (quantity, Hemoglobin)
- Possible immobilizations, application of tourniquet or hemostatic dressing, site of tourniquet, time of application
- Transport time to the hospital
- Death before admission to hospital, date and time of death

**Data collected at the hospital after admission:**

- Results of blood group prior to administration of FLYP
- Results of biological samples for hemostasis analysis before and after administration of FLYP: PT, APTT, fibrinogen, CRP, coagulation factors
- Results of arterial blood gas: lactate, PaO2 (FiO2 noted), if performed
- Clinical parameters at intake (HR, BP, RR, SpO2, Temperature, Hemoglobin), time of collection
- Complete lesion assessment, time of assessment
- Severity score: ISS, IGSII, revised trauma score, TRISS
- Possible hemostasis surgery, administration of tranexamic acid, type of surgery, time of day
- Blood products transfused: name of the components transfused, time of administration
- Transfused blood derivatives: fibrinogen, ..., time of administration
- Evolution of amine requirements, administered syringe flow rate, administration time
- Evolution of hemostasis parameters
- Respiratory evolution: ventilation parameters (duration of mechanical ventilation, FiO2, etc.), ARDS-type complications
- Length of stay in Intensive care unit, mortality, date and time of death
- Length of stay, in-hospital, and 30-day mortality.

# 4 SCREENING and ENROLLMENT

## 4.1 Inclusion Criteria

Adult victim of a hemorrhagic shock of traumatic origin with SBP <70 mmHg or SHOCK INDEX (HR/SBP) >1.1, at a given time of prehospital medical care.

The blood pressure and heart rate criteria do not need to be simultaneous.

## 4.2 Non-inclusion Criteria

- Refusal of the patient to participate in the research
- Patient not affiliated to a social protection system
- Age < 18 years
- Patient deprived of liberty
- Patient subject to a safeguard of justice measure
- Unrectified Legionnaire
- Pregnancy
- History of known allergies to amotosalen and psoralens
- Treatment by coagulation factor other than FLYP

- Patient in cardiac arrest

### 4.3 Exclusion Criteria

- Patients who did not receive the final blood sample (required for the primary endpoint)
- Refusal to participate by the patient

### 4.4 Procedure for transfusion reactions

- The participation of a patient in the study is voluntary. Any patient wishing to interrupt the experiment in which he is participating is free to do so at any time without having to justify it. The data collected will not be used in the analysis.

- The administration of the study treatment is stopped in the event of any adverse event (AE) (abnormal clinical manifestation,...), and the incriminated experimental treatment is retained. The usual management and corrective measures are continued in the field, during transport, and at the receiving hospital. A meeting of the independent monitoring committee is organized to discuss the discontinuation or continuation of the study depending on the nature of the AE.

In case of persistent consequences, the investigator will follow the patient as long as necessary.

### 4.5 Hospital care follow-up if the research is stopped

The medical care of the persons planned at the end of the research or in case of premature termination is carried out within the receiving hospital or in another hospital structure after transfer according to the usual care carried out in the care structures.

### 4.6 Arrangements for monitoring individuals involved in research based on, among other things, non-clinical data, including the duration of monitoring and follow-up procedures, and procedures for the medical management of individuals involved in research in the event of an emergency

In the event of an emergency, research subjects are managed and monitored by a prehospital and then hospital care team according to the department's usual protocols until they are discharged from the department (death, return home, other care facilities), and if necessary, until the resolution of the SAE.

### 4.7 Procedures for obtaining consent from research subjects

All selected patients who meet the inclusion criteria will be offered to participate in the study. They will be informed orally about the study and the different examinations performed by the prehospital or hospital investigating physician.

Consent must be obtained primarily from the person who is to be the subject of the research.
However, in view of article L. 1122-1-2 of the Public Health Code, if the urgency of the situation does not allow the consent of this person, it is requested from the family or the trusted person if they are present.
If consent could not be obtained prior to administration of the experimental treatment, the patient may be included by decision of the investigator, and consent will be sought from the patient as soon as his or her condition permits for the continuation of the research.

In the event that the patient dies after emergency inclusion, but before he or she is able to sign the consent to proceed, the data will be retained for the research. If possible, the investigator will inform the family.

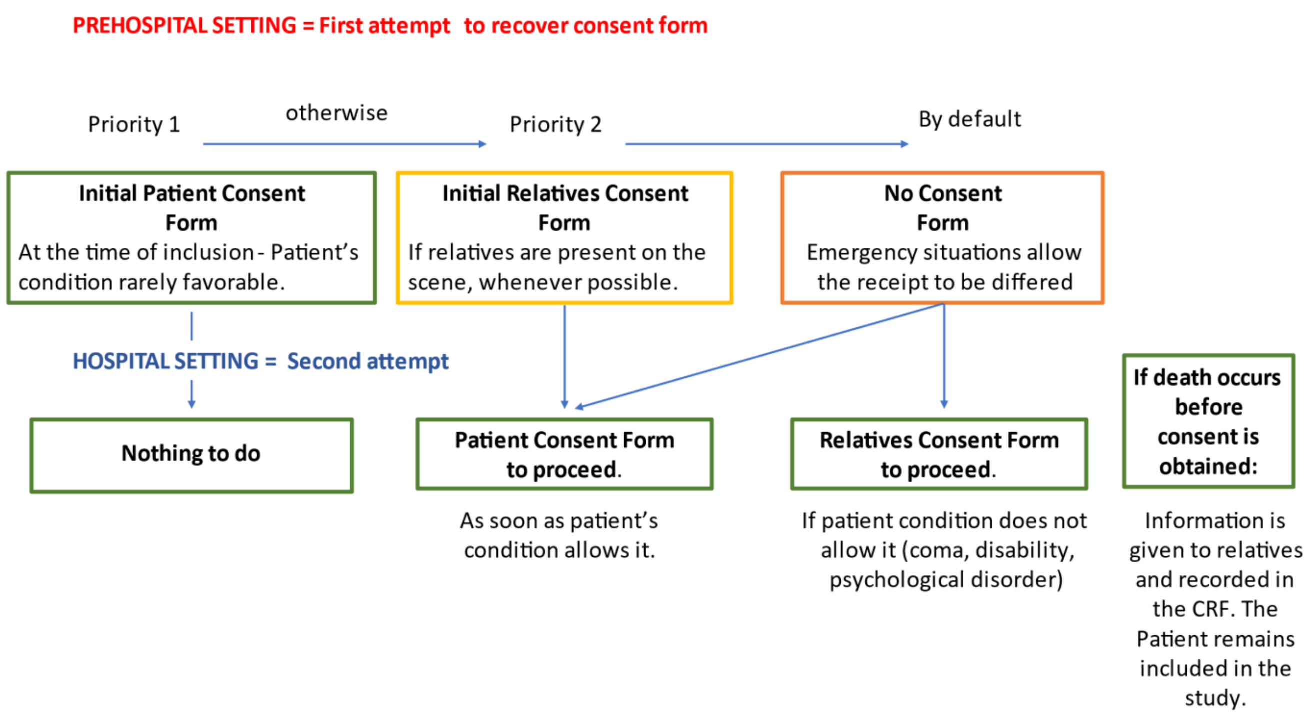


<u>Rules for the obtention of patient's consent, under the French legislation and approved by the ethics committee</u>

### 4.8 Concurrent participation in another research study and expected exclusion period at the end of the study

The patient is not a priori scheduled to participate in another interventional study during the time of study participation. There is no exclusion period.

## 5 TREATMENTS ADMINISTERED

### 5.1 FLYP

#### 5.1.1 Indications

Its indications are :
- moderate, poorly progressive or controlled hemorrhage (guided primarily by laboratory tests with a patient/control prothrombin time ratio > 1.5)
- hemorrhagic shock and situations at risk of massive bleeding.

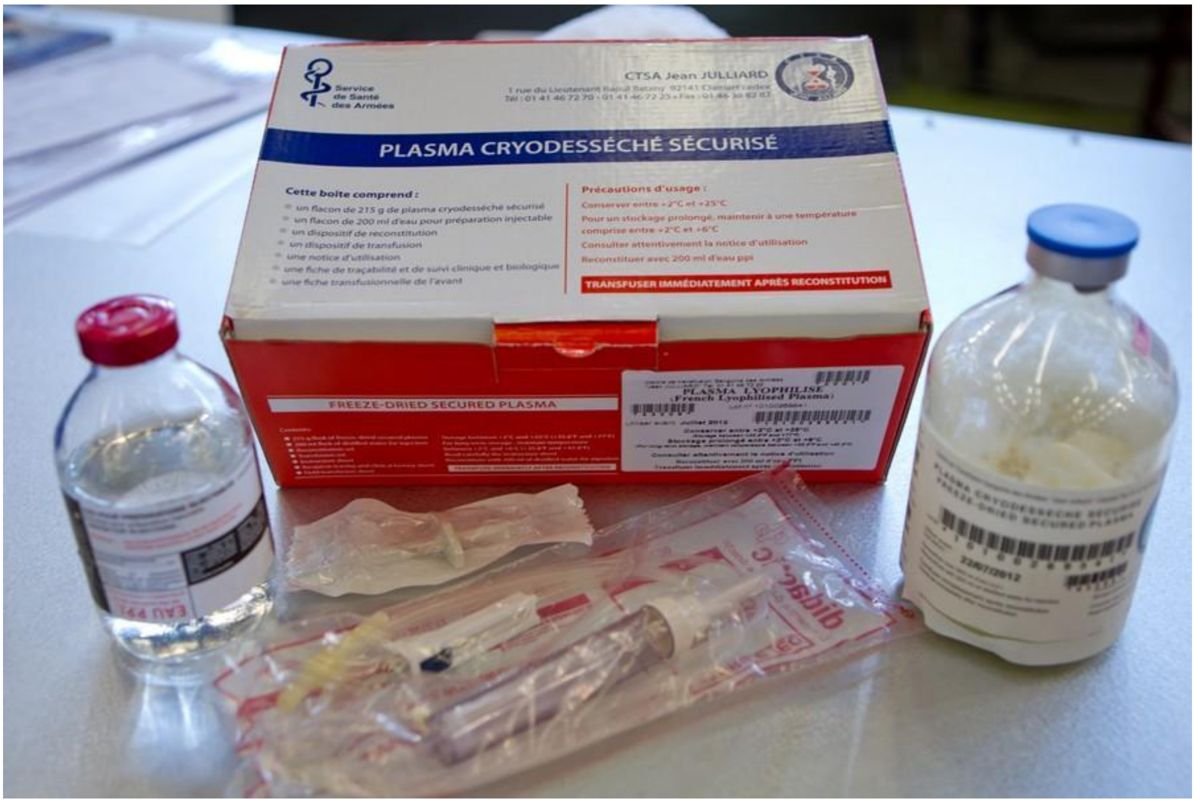


### 5.1.2 Packaging of the product

The freeze-dried plasma is secured by treatment with amotosalen.
It is vacuum-packed in a 200 ml bottle.
An instruction leaflet, a prescription-delivery-clinical and biological monitoring form systematically accompany the product in its packaging.

### 5.1.3 Conservation

FLYP must be stored in its packaging at a temperature of between +2°C and +25°C. Under these conditions, the maximum shelf life is two years.
The product can be used between 0 and 40°C.

Reconstitution at room temperature should be completed within six minutes. The resulting solution is practically free of suspended particles.
FLYP should be used no later than 6 hours after reconstitution.

### 5.1.4 Labels

- 1 Product identification label with lot number, expiration date,
- 3 Detachable labels indicating the lot number

### 5.1.5 Reconstitution

- Remove the protective cap from both units and disinfect the surface of each cap.
- First, **pierce the stopper of the water for the injection bottle with** one of the bevels of the transfer system, then pierce the stopper of the bottle containing the lyophilized with the free bevel of the transfer system; make sure that all of the water flows onto the powder by making a rotary movement to moisten the maximum surface of the powder.
- To facilitate reconstitution, gently shake the bottle by horizontal rotations so that the water touches all the powder without making too much foam (do not shake violently).

- Wait for 3 to 5 minutes for complete dissolution before injecting. FLYP appears as a mostly cloudy liquid, with occasionally visible particles that are not signs of spoilage.

#### 5.1.6 Transfusion

- Check that the glass unit is undamaged before transfusion. Under no circumstances should the contents of damaged units be infused.
- Transfuse intravenously immediately and in a single dose over 5 to 15 minutes per unit.

#### 5.1.7 Disposition of units

Any unused product will be returned to the CTSA.
Any used product or waste will be disposed of on-site in conformity with the hospital procedure (Incineration of hospital waste - DASRI).

#### 5.1.8 Plasma Traceability

Labels to detach from the bottle and stick on:

- the prescription form for the delivery and the clinical and biological follow-up of the Lyophilized plasma (to be returned to the CTSA)
- the medical clinical observation form (which contains the patient's source data)
- the CRF of the study

### 5.2 PHYSIOLOGICAL SERUM

#### 5.2.1 Indications

Prehospital severely injured patient, following the guidelines.

#### 5.2.2 Packaging and unit shape of the product

Flexible bag containing 500 ml

#### 5.2.3 Conservation

At room temperature

#### 5.2.4 Label

The label is put on the top of the kit "study bag," which contains the two saline bags. It contains the following items

- Name of the study
- Patient number
- Lot number

#### 5.2.5 Administration

The solution is ready to use.

### 5.3 Permitted and prohibited treatments in the protocol

All treatments usually used to manage an acute bleeding victim are allowed and should be noted in the observation book.

CAUTION: Do not administer clotting factors before the final blood sample for PT as this will render the observation unusable.
The administration of tranexamic acid is authorized and even planned on a systematic basis as soon as possible while respecting the contraindications. The dosage should be 1 gram in the prehospital setting.

### 5.4 Monitoring of the infusion/ transfusion

For each patient, the experimental treatment, the modalities, and the chronology of administration will be collected and noted on the medical clinical observation form and the CRF.

### 5.5 Practical procedures for accounting, storage, and delivery of experimental treatments within the investigating centers

The experimental treatments are packaged and supplied by the CTSA. They will be distributed in batches of 2 (1 FLYP and one normal saline), either by the CTSA for the BSPP or by the regional Etablissement Français du Sang-EFS (tripartite agreement between the CTSA, the regional EFS, and the recruiting center) for the other prehospital investigating centers.

Study treatments will be stored and accounted for at each center. A copy of the detailed delivery note (date of shipment, lot number, quantity, expiry date) of the CTSA shipments will be kept there (Appendix 2). In these centers, these treatments will be stored in the in-house pharmacies under the same conditions as the other treatments routinely used in the prehospital setting but in a space specifically dedicated to the study.

Only one experimental treatment (bag containing FLYP or not) will be made available to the prehospital team in the AR or in the SMUR.
This treatment will be taken out of its packaging only when it is used.

As soon as the physician comes back from the intervention, he must return all the study elements to the center's manager.
No vial of FLYP from an opened batch shall be present in any AR/SMUR.
Unused, expired or unused FLYP units will be redirected to the CTSA.
Any FLYP that has not expired but is not usable due to improper storage conditions or any other reason must also be returned to the CTSA (with details of nonconformities) for inspection.

Suppose the FLYP is destroyed by the user (degradation without the possibility of a return to the CTSA) or in case of loss. In that case, the delivery slip will be returned to the CTSA with the mention destruction or loss of product at the level of the batch number, a duplicate will be kept at the concerned recruiting center.

Used and empty bottles will be thrown away (Incineration of hospital waste - DASRI).

## 6 EVALUATION OF THE EFFECTIVENESS OF THE TREATMENT

### 6.1 Description of effectiveness evaluation parameters.

#### 6.1.1 Clinical parameters

During the patient's care at the intervention site, vital signs (BP, HR, and consciousness) are continuously monitored from the arrival of the first aid, then the arrival of the AR or the SMUR until the arrival at the hospital.

#### 6.1.2 Biological parameters

Improvement of biological parameters of hemostasis:
Variation between PT/INR at hospital admission and initial field PT/INR in the 2 FLYP(+) and FLYP(-) groups.

### 6.2 Planned methods and timing for measuring, collecting, and analyzing effectiveness metrics

Is responsible for the proper routing of blood tubes and the retrieval of results to be reported in the CRF:
- The prehospital co-investigator for the initial assessment
- The hospital co-investigator for the in-hospital assessment

The laboratory of each hospital will be asked to analyze the tubes.
Particular attention should be paid to the identification of samples and to the conservation of samples at room temperature.
Care should be taken to ensure that the time for analysis and determination of coagulation factors is less than 6 hours.

# 7 SAFETY ASSESSMENT

## 7.1 The vigilance during the trial

### 7.1.1 Definitions

✓ **Adverse event** (article R.1123-39 of the public health code)
Any adverse event that occurs in a person who is a subject of biomedical research, whether or not the event is related to the study or to the product to which the research relates.

✓ **Adverse reaction** (article R.1123-39 of the public health code)
Any adverse reaction in humans that is relevant to biomedical research and has been judged by the investigator to be possibly related to the use of the investigational product at any dose.

✓ **Adverse Receiver Effect:**
An adverse event is any occurrence in a recipient of labile blood products that is or may be related to the administration of a blood product.

✓ **Serious adverse reaction** (article R.1123-39 of the public health code and ICH guide E2B) = **adverse reaction** which :
- leads to death
- endangers the life of the person who is the subject of the research
- requires hospitalization or prolongation of hospitalization
- causes a significant or lasting disability or handicap
- results in a congenital anomaly or malformation
- or any event deemed medically serious by a health professional

✓ **Unexpected Serious Adverse Event** (article R.1123-39 of the Public Health Code) = adverse event whose nature, severity, or evolution do not correspond to the information contained in the Investigator's Brochure (IB), for an unauthorized investigational product or in the case of an authorized product, used under the conditions of the MA, the summary of product characteristics, SPC, Instructions for Use).

✓ **New security fact** (order of May 24, 2006)
New safety data that may lead to a reassessment of the benefit/risk ratio of the research, or that may be sufficient to consider changes in the research documentation, the conduct of the research, and, if appropriate, in the use of the product

## 7.2 Investigator's responsibilities

### 7.2.1 Notification of AE to the sponsor

The investigator should report all biological and clinical AEs in the CRF. and report them to the sponsor if necessary.

### 7.2.2 What should be declared?

- **Expected SAEs:**
    - **subject to immediate notification by the investigator:**
        - Allergic reactions
        - TACO (transfusion-associated circulatory overload)

In sporadic cases, transfusion of fresh frozen plasma may result in allergic reactions or respiratory complications. Therefore, it is important to observe whether the same clinical events occur with FLYP even though the method of manufacturing freeze-dried plasma reduces this risk.

- **not requiring immediate notification by the investigator**

Serious adverse events related to the natural and usual course of the disease and not related to the investigational treatment, nor the acts and procedures added by the research:

- Aggravation of hemorrhagic shock
- Any complications secondary to hemorrhagic shock
- Hospitalization for any length of time: patients suffering from hemorrhagic shock in a traumatic context will benefit from multidisciplinary care in the hospital, including intensive care, surgery, medicine, and functional rehabilitation.
- Death: as the death rate for severe trauma patients is around 50%; given the high activity of the receiving departments, the coordinating investigator must be notified of death via [redacted]sparis.fr within 72 hours. The coordinating investigator will transmit the information to the promoter (Research and Innovation [redacted]

- **Unexpected SAEs:**

Upon **arrival at the hospital,** the patient will be taken care of by the hospital's emergency or intensive care unit, and a follow-up of the initial workup will be performed.

- After the extension workup, a prognosis can be given. Any unexpected worsening of the patient's existing condition that is not consistent with the prognosis given by the initial extension workup should be considered an SAE and reported by the hospital investigating physician.

- **The occurrence of in utero exposure**

Any pregnancy in which the fetus (from pre-embryonic stage to birth) may have been exposed at any time to FLYP, even if not associated with an adverse event, should be reported to the sponsor without delay.

#### 7.2.3 When to report?

Immediately and no later than 24 hours after the investigator becomes aware of an SAE.

The investigator ensures that relevant information regarding the follow-up is communicated to the sponsor within seven days.

Any SAE must be reported, if it occurs for a research participant, during the entire duration of the research, i.e., from the date of signature of the consent form until the end of the participant's follow-up, i.e., 30 days after inclusion in the protocol, or at the patient's death if this occurs during the follow-up period.
Once the trial is completed, the investigator is not required to actively follow participants for adverse events. After the end of follow-up, serious adverse events experienced by a participant_will still be reported to the sponsor if the investigator is aware of them.

#### 7.2.4 How to report?

The initial notification is reported in writing and in detail using the "Initial Serious Adverse Event Report" form (located in the appendix of the CRF).

The initial notification must contain, at a minimum, all of the following:

- an identifiable participant code
- an identifiable notifier
- the treatment of the test (FLYP(+)/ normal saline)
- the adverse event (date of occurrence, intensity, relationship to the research)

Initial notification is followed by detailed written follow-up reports using the “Serious Adverse Event Follow-up Report” form (located in the appendix of the CRF.) to monitor the case’s progress or complete the information.

The investigator completes and sends the dated and signed SAE. report form, as well as any relevant anonymized document related to the SAE (e.g., laboratory results, examination or hospitalization reports...) to :

- **Regarding Hemovigilance:**
  - These events must be declared without delay:
    - **to the CTSA** [redacted]

- **Concerning pharmacovigilance**

It does not concern FLYP, which is a labile blood product. However, for the proper conduct of the study, pharmacovigilance is set up for any product that does not directly concern FLYP.

- **to** [redacted]

In the case of in utero exposure, the investigator completes the “Follow-up form for a pregnancy discovered during biomedical research” and faxes it to Hemovigilance.
The investigator must follow the pregnant woman until the end of the pregnancy or its termination and notify the sponsor of the outcome with this form. If the outcome of the pregnancy falls within the definition of serious adverse events (spontaneous abortion, t_ermination of pregnancy, fetal death, congenital anomaly, etc.), the investigator must follow the procedure for reporting SAEs.

### 7.3 Sponsor’s responsibilities

The sponsor is responsible for reporting to the ANSM and the CPP (Ethic Committee) any serious and unexpected adverse events attributable to the labile blood cell therapy product and/or the protocol within 15 days (7 days in the case of death and life-threatening events).

On the anniversary date of the first inclusion, the sponsor prepares a safety report including:
- the list of potential research drug product-related SAEs
- a concise and critical analysis of the safety of the participants involved in the research.

This report is sent to the ANSM and the CPP (Ethic Committee) within 60 days of the anniversary of the first inclusion.

### 7.4 Planned methods and timing for measuring, collecting, and analyzing safety assessment metrics

**During the prehospital phase:**
A permanent presence of an emergency physician and a nurse with the person undergoing research. Continuous surveillance of the clinic, continuous monitoring of the constants: HR, BP, SpO2. Verification of the absence of signs of drug intolerance.
Collection of the different parameters in the prehospital CF.

The occurrence of adverse event(s) or the aggravation of adverse event(s) in the prehospital phase will be reported by the physician who managed the patient in the context of prehospital management.

**On admission to the hospital:**
Management by the emergency department or intensive care unit and implementation of departmental protocols for the management of patients in traumatic hemorrhagic shock; monitoring of the clinic and vitals (HR, BP, SpO2) Verification of the absence of signs of drug intolerance.
Collection of the different parameters in the hospital CRF.

The occurrence of an unexpected event or worsening of an unexpected event in the hospital phase will be reported by the department in charge of the patient.

### 7.5 Data Safety Monitoring Board (DSMB/ CSI)

An oversight committee made up of members independent of the project will be set up:

[redacted]
Department of anesthesia and intensive care
[redacted]
Hematology-Transfusion Unit
[redacted]

This committee is informed during the study of any AE/SAE related to the administration of FLYP.
He is informed, in writing, by the promoter, and the information relating to the follow-up of the AEs will be transmitted to him.

It will be responsible for ruling on the causality of AEs reported by investigators during the trial. It has an advisory function when called upon by the sponsor on medical issues such as tolerance and adverse events.

The committee will decide whether or not to stop the study and whether or not to discharge the patient from the study. The recommendations concerning the continuation, possible modifications, or premature termination of the study will be transmitted to the sponsor.

This committee meets once every six months, except in particular cases.

### 7.6 Reporting of Adverse Events

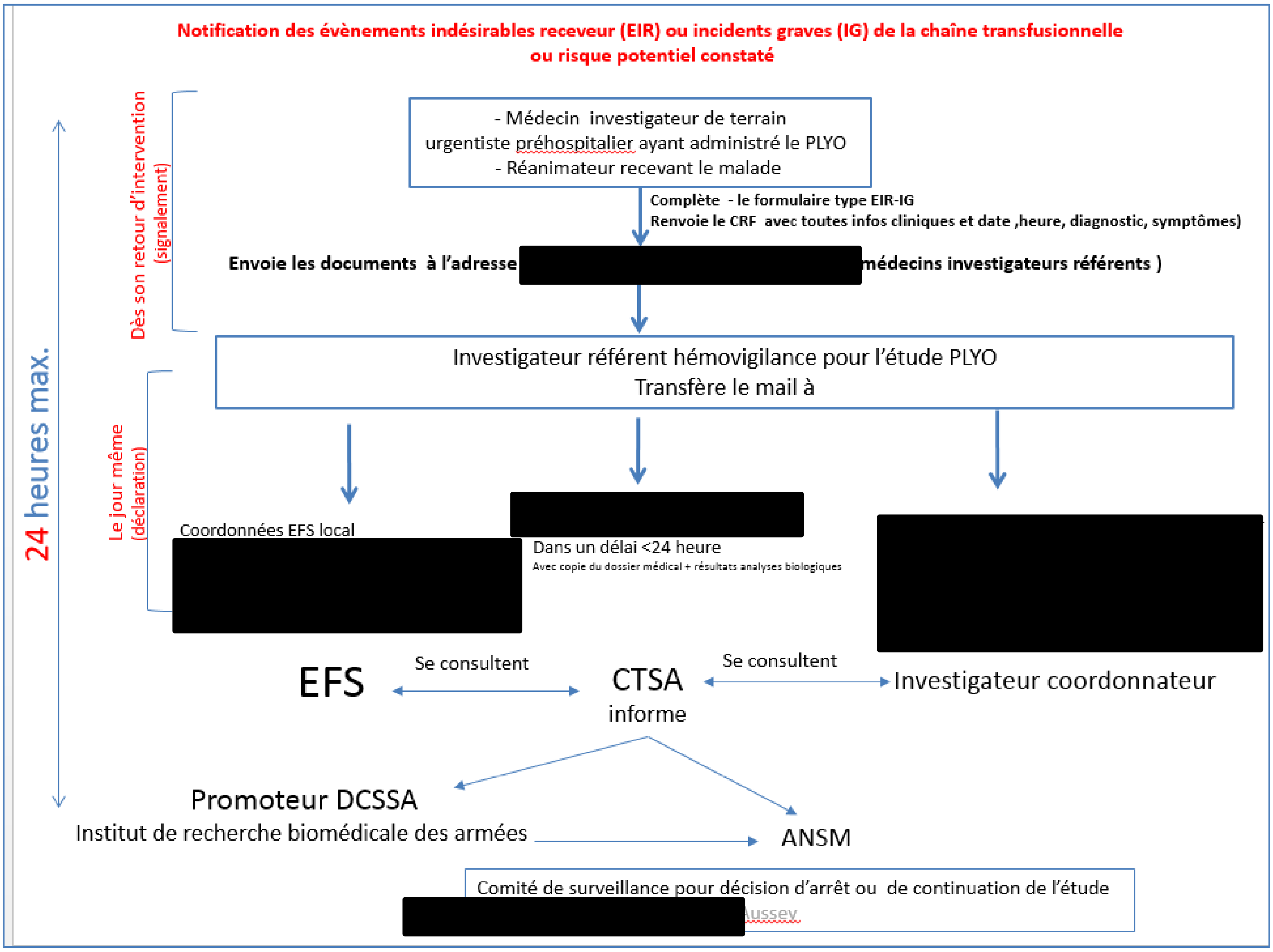
Notification des évènements indésirables receveur (EIR) ou incidents graves (IG) de la chaîne transfusionnelle ou risque potentiel constaté
24 heures max.
Dès son retour d'intervention (signalement)
- Médecin investigateur de terrain urgentiste préhospitalier ayant administré le PLYO
- Réanimateur recevant le malade
Complète - le formulaire type EIR-IG
Renvoie le CRF avec toutes infos cliniques et date ,heure, diagnostic, symptômes)
Envoie les documents à l'adresse [redacted] médecins investigateurs référents )
Le jour même (déclaration)
Investigateur référent hémovigilance pour l'étude PLYO
Transfère le mail à
Coordonnées EFS local
[redacted]
[redacted]
Dans un délai <24 heure
Avec copie du dossier médical + résultats analyses biologiques
[redacted]
EFS
Se consultent
CTSA
informe
Se consultent
Investigateur coordonnateur
Promoteur DCSSA
Institut de recherche biomédicale des armées
ANSM
Comité de surveillance pour décision d'arrêt ou de continuation de l'étude
[redacted]

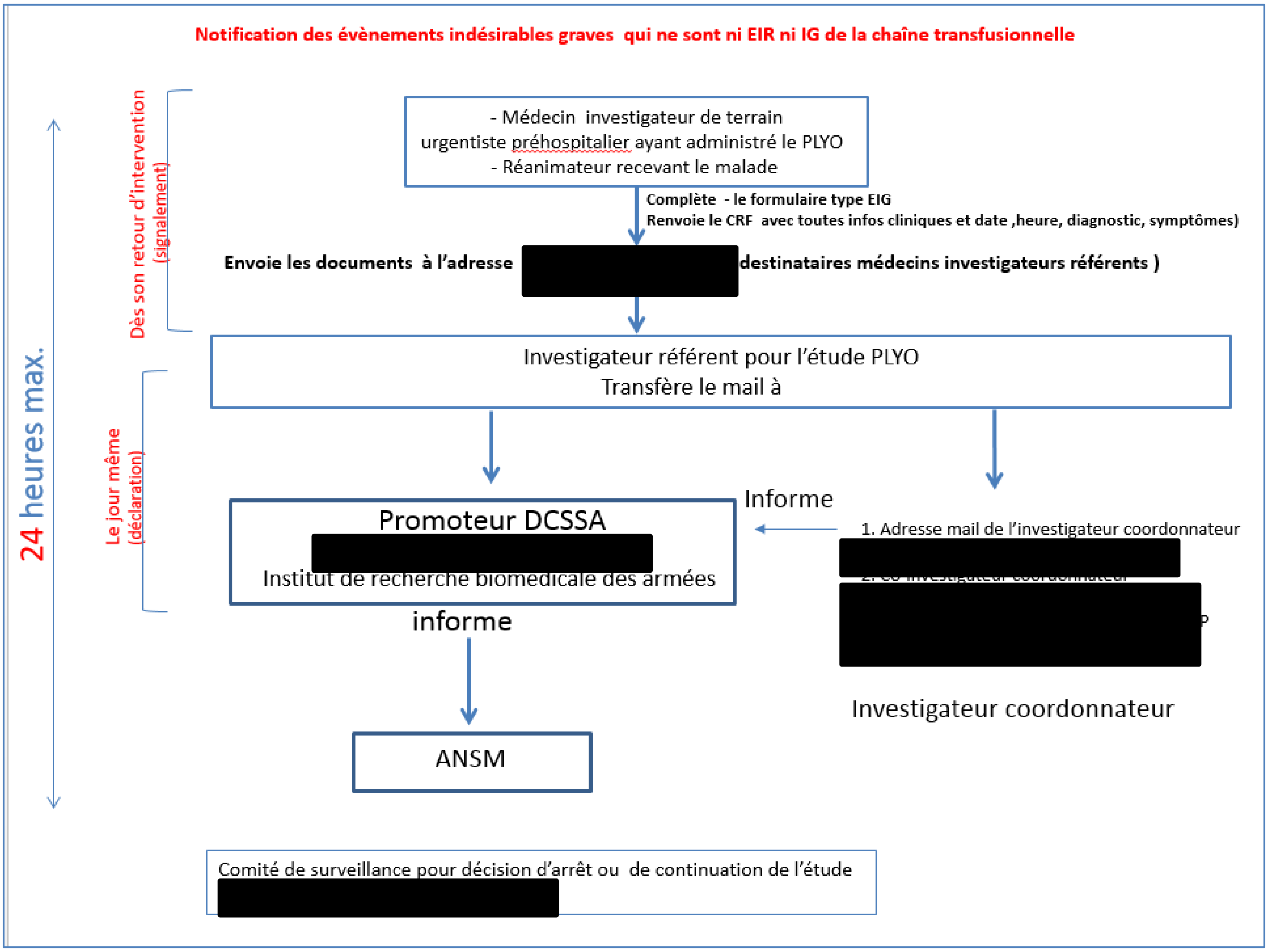


# 8 STATISTICS

## 8.1 Analysis plan

The complete statistical analysis plan is the subject of a separate document.

We will report categorical variables as frequencies (%) and use Fisher's exact test to compare. Continuous variables will be expressed as medians (IQR) and will be compared using the Mann–Whitney U test. The effect sizes will be represented by the median difference (95% CI) for continuous variables and the risk ratio (95% CI) for categorical variables. All comparisons will use two-sided tests, and a P value of less than 0.05 will be considered to indicate statistical significance. We will not use any correction to account for multiple comparisons.

**A comparison of key patient characteristics will be performed to validate the comparability of the two groups.**
This comparison will be made for the main variables of interest :
Age, sex, comorbidities, type of injury, mechanism of injury, anatomic localisation of trauma, time response of advanced life support teams, vital status at the point of injury (blood pressure, heart rate, shock-index,oxygen saturation), prehospital biological measures (Prothrombin time and INR value, Hemoglobin and lactic acid concentrations), body temperature, basic life support therapeutics, advanced life support therapeutics, cumulated crystalloid volume, cumulated plasma volume, prehospital red cell units transfused, vital signs on arrival at the intensive cae unit (blood pressure, heart rate, shock-index, injury severity score, traumatic brain injury confirmed with CT scan, laboratory values - Hemoglobin, platelets, lactic acid, base excess)

The analysis will take into account a possible center effect

**Data Analysis for Primary Endpoint:**

- For the primary outcome analysis, INR at hospital admission will be considered to have been performed at a comparable time in each patient and will be compared across the two arms with the Mann–Whitney U test. If the initial characteristics of patients are unbalanced between the two groups after randomization, an adjusted analysis will be considered with a regression method.
- When appropriate, both adjusted and unadjusted results will be reported.

**Data Analysis for the Secondary Endpoints:**

- The prehospital INR delta [between INR- management at the procedure site and INR-on arrival at the hospital] between the two groups will be studied with multilevel logistic regression modeling that will be fitted using a generalized estimating equation method to take into account time and both patient and hospital clustering. Where appropriate, this regression might be adjusted for unbalanced variables.

- The mean difference in fibrinogen levels between the two groups will be compared by a GEE model in the same way as for INR.

- The transfusion requirement will be compared by the number of blood products and plasma units transfused after arrival at the hospital, including packed RBCs, platelet concentrates, fibrinogen, coagulation factors, and Fresh frozen plasma. The transfusion requirement will be assessed at 6 and 24 h, taking the investigating center into account as a stratifying variable.

- The comparison of the median number of RBCs transfused between the two groups will use the Mann–Whitney U test

- The median number of days in the intensive care unit between the two groups will be studied overall and among surviving patients. They will be compared between the two groups by a Mann–Whitney U test.

- For each arm, actuarial survival will be visualized using the Kaplan–Meier estimator and compared with the log-rank test to estimate the effect of FLYP on survival. A multivariable Cox proportional hazard regression model may be fitted in cases of potential imbalance.

- The feasibility of administering FLYP will be judged by the responses to the CRF. page 9
- (Rate of positive responses to the various items)

- Safety outcome: The adverse event incidence will be compared between the two groups, using a Chi2 or a Fisher exact test.

- Exploratory subgroup analysis: we will report the effect size on the INR value in subgroups defined by variables of interest such as the type of injury, the prehospital vasopressor use, the prehospital intubation, the presence of a brain injury.

- Post-hoc analyses to further explain the primary outcome.

We will measure, with an adapted model, the relationship between the INR value and the ratio of plasma-to-crystalloid volume, which seems to be an indicator of plasma dilution by superadded serum

## 8.2 Anticipated number of people to be included in the research with its statistical justification and anticipated number of people in each research location

- The recent literature [4] reports a coagulopathy rate of 50% of severely injured patients on arrival at the hospital.
-

We determined that a sample size of 30 patients with coagulopathy in each group was adequate to detect a mean difference of 0.3 in INR values at hospital admission, assuming a standard deviation of 0.4, with a two-sided type I error of 0.05 and a power of 80%.

We chose this 0.3 anticipated effect size pragmatically, based on clinical judgment and the results of one previous observational study [16] in patients with traumatic hemorrhage of “before-after” FLYP administration

with Prothrombin Time (PT) as the endpoint, showed a significant improvement in PT "after" FLYP. A total of 124 patients were required to observe a statistically significant difference.

Assuming an incidence of coagulopathy of 50%, this resulted in 60 subjects per group. Given the a priori loss of 10% due to lack of informed consent, we rounded the target size of each group to 70 and included a total of 140 patients. To consider a median difference of 0.3 in INR values instead of mean difference, the sample size needs to be approximately 25 coagulopathic patients per group, for a total of 120 patients, based on the same alpha and beta risk assumptions.[17] INR values will be presented according to their median (IQR) and by a three-category variable with cutoff values of 1.2 and 1.5.

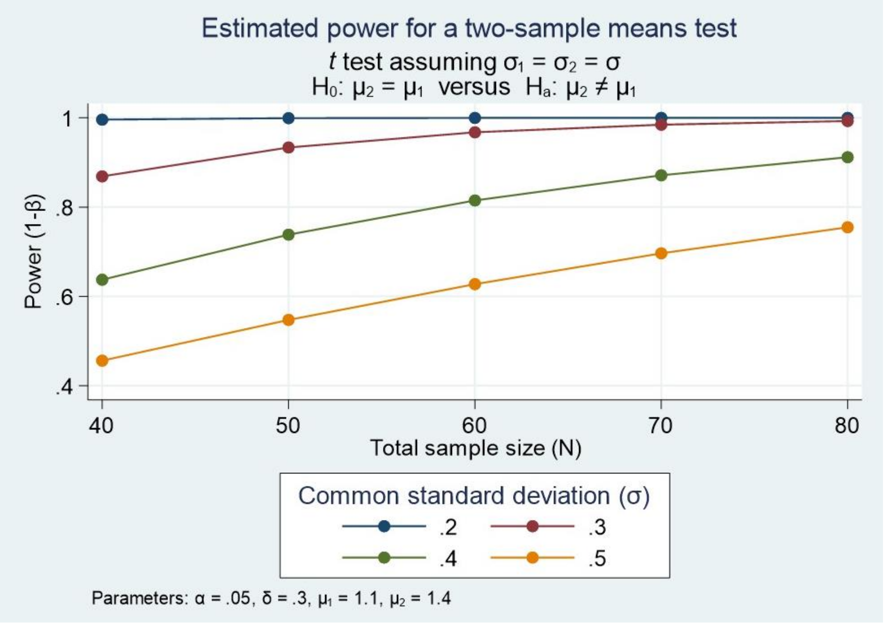


### 8.3 Statistical significance

The tests are two-sided, with the significance level set at 0.05. The statistical analysis will be conducted using STATA 14.0 or an updated version

### 8.4 Statistical analysis for stopping the research

No interim analysis is planned.

### 8.5 Missing or invalid data

Multiple imputation procedures will be performed on the data of patients involved in the principal analysis. We will address the problem of missing data using the state-of-the-art multiple imputation techniques, including all analysis variables, under the assumption that missing values are missing at random. Multiple imputation procedures will be conducted using the chained equation. We will estimate the fraction of missing information and apply the multiple imputation procedure, generating at least 50 imputed datasets. Analyses will run on each dataset and be pooled according to Rubin's rules.[18] We will compare the distributions of the observed, imputed, and completed values, and the post-imputation diagnostic plots will report the observed and completed values. We will use the kernel- Epanechnikov density estimator for continuous variables and a histogram for categorical variables. In addition, we will use the Kolmogorov- Smirnov statistics to compare the observed, and imputed distributions of each continuous imputed variable The null hypothesis (i.e., observed and imputed data follow the same distribution) is rejected at P<0.05. The imputed results will be presented as main results if they don't differ from the complete case analysis estimates.

### 8.6 Substantive modifications to the research protocol

If there is a need for changes to the original strategy analysis plan, a modification will be submitted to the CPP (Ethic Committee) in advance.

### 8.7 Patients included in the analysis

Analysis populations will be :

- in intention-to-treat for the safety outcome (ITT: taking into account all randomized patients)
- in modified intention-to-treat analysis for the primary outcome (mITT: taking into account all randomized patients except those for which the eligibility criteria were not met)
- in Per-Protocol (PP: taking into account patients randomized but excluding the patients for which the eligibility criteria were not met and those who were declared free from any haemorrhagia after the hospital check-up.

All statistical analyses will be performed with STATA® software 14.0 or an updated version for Windows (STATACORP LLC, College Station, Texas, USA).

## 9 RIGHT OF ACCESS TO SOURCE DATA AND DOCUMENTS

The sponsor has obtained the agreement of all parties involved in the research to ensure direct access to all research sites, source data, source documents, and reports for quality control and audit purposes by the sponsor.

The investigators will make available the documents and individual data strictly necessary for the follow-up, quality control, and audit of the biomedical research to the persons mandated by the sponsor following the legislative and regulatory provisions in force (articles L.1121-3 and R.5121-13 of the public health code).

Any original document or object that proves the existence or accuracy of a data or fact recorded during the research is defined as a source document.

Access to data will be limited to those who need access to perform their tasks.

### 9.1 Privacy and Confidentiality

Following the legislative provisions in force (articles L.1121-3 and R.5121-13 of the Public Health Code), persons with direct access to the source data will take all necessary precautions to ensure the confidentiality of information relating to the investigational medicinal products, to the research, to the persons involved, and in particular with regard to their identity and the results obtained. Furthermore, these persons, as well as the investigators themselves, are subject to professional secrecy.

During the biomedical research or at its conclusion, the data collected on the persons who lend themselves to it and transmitted to the sponsor by the investigators (or any other specialized intervening parties) will be made anonymous. Under no circumstances must the names of the persons concerned or their addresses appear in clear text.

The sponsor will ensure that each person who participates in the research has given written consent for access to their data that is strictly necessary for the quality control of the study.

If the participant is included on an emergency basis, in the absence of signed consent (from the family, the trusted person, the patient in the case of consent to continue the study), the principal investigating physician at each center will answer questions from personnel mandated by the sponsor for monitoring, based on the medical record. Direct access to medical data by persons mandated by the sponsor will not be possible without the signature of a consent form.

## 10 QUALITY CONTROL AND QUALITY ASSURANCE

The medical observations of the prehospital team will be kept on the patient's "medical observation sheet". The data concerning the study will be reported in the observation notebooks provided for the study, according to good clinical practices, taking into account the different steps of the patient's management in the protocol. Any deviation from the protocol will be reported, as well as the reason for it. The data collection must be exhaustive and will be regularly checked by a clinical research associate (C.R.A.) according to the planned procedures.

The monitoring of the trial will be carried out by CRAs mandated by the sponsor. It will depend on the number of enrolments in the study.

During site monitoring visits, CRAs should be able to consult:

* the investigator's binder containing the consents of the participants or their relatives
* the data collection books of the patients included
* patient medical observation sheets and any other source documents
* The hospital information system

The monitoring will check at least the following points:

* the existence of patients, the consent process
* compliance with inclusion and non-inclusion criteria
* the primary endpoint
* monitoring and reporting of SAEs
* the occurrence of new facts requiring the tabling of an amendment

A written monitoring visit report will be prepared for each visit and kept in the sponsor's documentation.

At the end of the trial, closing procedures will be applied, with all documents and source data filed. Once the final analysis is completed and validated, the entire file and data are sealed and archived according to specific procedures in a secure facility.

In addition, investigators agree to accept quality assurance audits by the sponsor and inspections by the Competent Authorities. All data, documents and reports may be subject to regulatory audits and inspections without prejudice to medical confidentiality.

## 11 ETHICAL CONSIDERATIONS

The sponsor and the investigators undertake that this research will be carried out in accordance with the law n°2004-806 of August 9, 2004, as well as in accordance with the Good Clinical Practices (ICH version 4 of May [1,] 1996, and decision of November 24, 2006) and the Helsinki declaration.

The investigator is responsible for the conduct of the trial. He/she agrees to:

- to retain the source data and administrative documents related to the protocol,
- not to include volunteers before receiving official authorizations from the CPP (Ethic Committee) and the ANSM
- to respect the protocol
- to conduct the study in accordance with the moral, regulatory, ethical, and scientific principles that govern clinical research,
- to collect the informed and written consent of each volunteer,
- to report any serious adverse events.

Subjects will be given complete oral and written information about the trial. A letter of information will be given to the patient by the investigator or the physician who represents him/her prior to his/her inclusion in the study. The information letter and consent form will be prepared in triplicate, and one copy will be given to the subject; a copy will be kept by the investigator, who will forward the last copy to the sponsor in a sealed envelope for filing in the permanent trial documentation.

The consent will be signed by the investigator or the physician representing him/her and the patient and/or, if applicable, the trusted support person or a family member.

ANSM authorization and CPP (Ethic Committee) opinion

The sponsor will submit a request to the ANSM to obtain an approval from the CPP (Ethic Committee) before the start of the research, following article L1121-4 of the Public Health Code.

Changes to the protocol

The sponsor alone is authorized to modify the protocol in conjunction with the coordinating investigator.

Substantial modifications are those that have a significant impact on any aspect of the research, in particular on the protection of persons, including their safety, on the conditions of validity of the study, the quality and safety of the products tested, on the interpretation of the scientific documents that support the conduct of the research, or on how the research is conducted.
A request for substantial modification is sent by the sponsor either to the ANSM, or to the CPP (Ethic Committee), or to both of these organizations, as the case may be, for approval. Upon receiving the approval, the amended version of the protocol is sent to all investigators by the sponsor.

A non-substantial amendment to the protocol is a minor change or clarification that does not affect the conduct of the trial. These modifications will not be submitted to the competent authorities but will be agreed upon between the sponsor and the investigator and will be clearly documented (in the study follow-up file).

## 12 DATA PROCESSING AND RETENTION OF DOCUMENTS AND RESEARCH DATA

Data processing will be carried out under the conditions of confidentiality defined by the amended law of January 6, 1978, relating to the French data regulatory agency (CNIL). Data processing will be carried out in accordance with the requirements of the CNIL MR001 reference methodology.

The data concerning this study will be archived for a minimum of 30 years from the end of the research or its early termination without prejudice to the legislative and regulatory provisions in force.

The following documents related to this research are archived in accordance with Good Clinical Practices for a period of 30 years following the end of the research:

* By investigators:
  - The protocol and any amendments to the protocol
  - Observation books (copies)
  - Source records of participants who have signed a consent
  - All other documents and correspondence related to the research
  - Original signed informed consents from participants

All of these documents are the responsibility of the investigator for the duration of the regulatory archiving period.

* By the sponsor:

- The protocol and any amendments to the protocol
- The original observation books
- All other documents and correspondence related to the research
- A copy of the signed informed consents of the participants in a sealed envelope
- Documents related to severe adverse events

All of these documents are the responsibility of the sponsor for the duration of the regulatory archiving period.

No removal or destruction will be made without the agreement of the sponsor. At the end of the regulatory archiving period, the sponsor will be consulted for destruction.

All data, documents, and reports will be subject to audit or inspection.

The study site investigators and members of their research team will make a reasonable effort to ensure the research subjects' confidentiality.

## 13 FINANCING AND INSURANCE

The promoter Service de Santé des Armées (French Health Defence) provides funding of [redacted]

The insurance is taken by the French Health Defence

## 14 References


1. Boutonnet M, Pasquier P, Salvadori A, Auroy Y, Tourtier J-P. Advocacy to extend the use of continuous noninvasive hemoglobin measurement: Crit Care Med 2011;39(12):2783–4.

2. Pasquier P, Boutonnet M, Giraud N, Salvadori A, Tourtier J-P. Hypotension Redefined, Shock Index and Massive Transfusion. J Trauma Inj Infect Crit Care 2011;71(3):784–5.

3. Brohi K, Singh J, Heron M, Coats T. Acute Traumatic Coagulopathy: J Trauma Inj Infect Crit Care 2003;54(6):1127–30.

4. Floccard B, Rugeri L, Faure A, et al. Early coagulopathy in trauma patients: An on-scene and hospital admission study. Injury 2012;43(1):26–32.

5. Brohi K, Cohen MJ, Ganter MT, Matthay MA, Mackersie RC, Pittet J-F. Acute Traumatic Coagulopathy: Initiated by Hypoperfusion: Modulated Through the Protein C Pathway? Ann Surg 2007;245(5):812–8.

6. Glassberg E, Nadler R, Gendler S, et al. Freeze-Dried Plasma at the Point of Injury: From Concept to Doctrine. Shock 2013;40(6):444–50.

7. Borgman MA, Spinella PC, Perkins JG, et al. The Ratio of Blood Products Transfused Affects Mortality in Patients Receiving Massive Transfusions at a Combat Support Hospital. J Trauma Inj Infect Crit Care 2007;63(4):805–13.

8. de Biasi AR, Stansbury LG, Dutton RP, Stein DM, Scalea TM, Hess JR. Blood product use in trauma resuscitation: plasma deficit versus plasma ratio as predictors of mortality in trauma (CME): PLASMA USE IN TRAUMA RESUSCITATION. Transfusion (Paris) 2011;51(9):1925–32.

9. Martinaud C, Tourtier J-P, Pasquier P, Ausset S, Sailliol A. The French Freeze-Dried Plasma. J Trauma Inj Infect Crit Care 2011;71(4):1091–2.

10. Martinaud C, Ausset S, Deshayes AV, Cauet A, Demazeau N, Sailliol A. Use of Freeze-Dried Plasma in French Intensive Care Unit in Afghanistan. J Trauma Inj Infect Crit Care 2011;71(6):1761–5.

11. Sailliol A, Martinaud C, Cap AP, et al. The evolving role of lyophilized plasma in remote damage control resuscitation in the French Armed Forces Health Service: Evolving Role of Lyophilized Plasma. Transfusion (Paris) 2013;53:65S-71S.

12. Martinaud C, Civadier C, Ausset S, Verret C, Deshayes A-V, Sailliol A. *In Vitro* Hemostatic Properties of French Lyophilized Plasma. Anesthesiology 2012;117(2):339–46.

13. Sailliol A, Ausset S, Peytel E. La transfusion en situation d’exception, expérience du service de santé des armées. Transfus Clin Biol 2010;17(5–6):279–83.

14. Lee L, Moore EE, Hansen KC, Silliman CC, Chandler JG, Banerjee A. It’s not your grandfather’s field plasma. Surgery 2013;153(6):857–60.

15. Tourtier J-P, Palmier B, Tazarourte K, et al. The concept of damage control: Extending the paradigm in the prehospital setting. Ann Fr Anesth Réanimation 2013;32(7–8):520–6.

16. Cauet A. Transfusion de Plasma Lyophilisé : Données de l’Hémovigilance Active. Etude Observationnelle à l’Hôpital Médico-Chirurgical Kaboul International Airport 2010-2011. 2012;

17. O’Keeffe AG, Ambler G, Barber JA. Sample size calculations based on a difference in medians for positively skewed outcomes in health care studies. BMC Med Res Methodol 2017;17(1):157.

18. Rubin DB, editor. Multiple Imputation for Nonresponse in Surveys [Internet]. Hoboken, NJ, USA: John Wiley & Sons, Inc.; 1987 [cited 2021 September 5]. Available from: http://doi.wiley.com/10.1002/9780470316696

19. David J-S, Voiglio E-J, Cesareo E, et al. Prehospital parameters can help to predict coagulopathy and massive transfusion in trauma patients. Vox Sang 2017;112(6):557–66.

20. Beynon C, Erk AG, Potzy A, Mohr S, Popp E. Point of care coagulometry in prehospital emergency care: an observational study. Scand J Trauma Resusc Emerg Med 2015;23(1):58.

21. David J-S, Levrat A, Inaba K, et al. Utility of a point-of-care device for rapid determination of prothrombin time in trauma patients: A preliminary study. J Trauma Acute Care Surg 2012;72(3):703–7.

22. Garrigue D, Godier A, Glacet A, et al. French lyophilized plasma versus fresh frozen plasma for the initial management of trauma-induced coagulopathy: a randomized open-label trial. J Thromb Haemost 2018;16(3):481–9.

23. Sperry JL, Guyette FX, Brown JB, et al. Prehospital Plasma during Air Medical Transport in Trauma Patients at Risk for Hemorrhagic Shock. N Engl J Med 2018;379(4):315–26.

24. Smith IM, Crombie N, Bishop JR, et al. RePHILL: protocol for a randomised controlled trial of prehospital blood product resuscitation for trauma: Prehospital blood products for trauma-RCT protocol. Transfus Med 2018;28(5):346–56.

25. Ramagopalan SV, Skingsley AP, Handunnetthi L, et al. Prevalence of primary outcome changes in clinical trials registered on ClinicalTrials.gov: a cross-sectional study. F1000Research 2014;3:77.

26. Yuan Q, Yu J, Wu X, et al. Prognostic value of coagulation tests for in-hospital mortality in patients with traumatic brain injury. Scand J Trauma Resusc Emerg Med 2018;26(1):3.

**Prehospital Lyophilized Plasma for Trauma-Induced Coagulopathy in Patients at Risk**

**for Hemorrhagic Shock: A Multicentre Randomized Open-label Trial.**

**The PREHO-PLYO Study.**

## 15 LIST OF AMENDMENTS

| List of the PREHO PLYO Study protocol amendments from 2016 to 2019 | Ethics Committee |
|---|---|
| **Simplification of the inclusion criteria : Shock Index (Heart Rate/ Systolic Blood Pressure)** | 04/26/2016 |
| Addition of 4 Investigators | 04/26/2016 |
| **Prehospital Point of Care use by Advanced life support teams** | 01/10/2017 |
| Addition of 2 Investigators (Lyon, Annecy Hospital) | 01/31/2017 |
| **Addition of Consent Details (for the relative of a dead patient)** | 01/31/2017 |
| Addition of 5 Investigators (SMUR 75) | 01/04/2019 |
| **Shock index change from 1.3 to 1.1** | 11/03/2017 |
| Addition of 1 Investigator (SAMU 94) | 01/30/2018 |
| Addition of 2 Investigators (SAMU 13) | 11/08/2018 |
| Additional of 3 investigators (BSPP) | 11/08/2018 |
| **Extension of the study to September 2019** | 11/08/2018 |
| Change of coordinator investigator (HEGP hospital) | 11/08/2018 |
| Withdrawal of 1 investigator (Dead) | 01/15/2019 |
| Change of coordinator investigator from J.P Tourtier to D. Jost | 08/16/2018 |
| **Clarification of consent in emergency situation** | 03/12/2019 |
| **Change of primary endpoint from delta-INR to INR** | 07/12/2019 |
| Addition of Brest center (SAMU 29) | 01/15/2019 |

# Prehospital Lyophilized Plasma for Trauma-Induced Coagulopathy in Patients at Risk for Hemorrhagic Shock: A Multicentre Randomized Open-label Trial.

# The PREHO-PLYO Study.

## 16 STATISTICAL ANALYSIS PLAN.

### Version V6, May 27, 2019.

**Trial Title**

- Prehospital Lyophilized Plasma for Trauma-Induced Coagulopathy in Patients at Risk for Hemorrhagic Shock: A Multicentre Randomized Open-label Trial.

**Trial registration number**

- DCSSA 2014RC04
- ID RCB 2015-A00866-43

**SAP Version**

- Version SAP of May 27, 2019

**Protocol Version**

- This document has been written based on information contained in the study protocol Version dated May 27, 2019

**SAP revisions**

- First simplification of the inclusion criteria ( Date changed, 04/26/2016)
    - Deleting blood pressure and heart rate threshold values and using the Shock Index (ratio of heart rate to systolic blood pressure) with a threshold at 1·3
- Second simplification of the inclusion criteria (Date changed, 11/03/2017)
    - Change of the shock index threshold from 1·3 to 1·1

- Modification of Primary Endpoint ( Date changed, 07/12/2019)
    - Deleting "INR differential between pre-and in-hospital values" and replacing with the "INR value at hospital arrival."

The SAP versions were amended at the same time as the protocol

**Roles and responsibilities of SAP contributors**

- SAP Redactors : Daniel Jost, Julie Trichereau
- Senior statistician responsible: Julie Trichereau
- Chief investigator/clinical lead: Daniel Jost, Jean-Pierre Tourtier.

## 16.1 Background and rationale

- Acquired failures of hemostasis during severe bleeding promote and maintain bleeding. Lyophilised plasma (FLYP) has been administered many times in the military setting, but only non-randomized observational studies have been performed in this context. Furthermore, it has never been used in the civilian prehospital setting. Therefore, there are no prehospital studies available that have compared the currently recommended treatment of hemorrhagic shock without FLYP versus with FLYP.

## 16.2 Objectives

- Hypothesis
  - The null hypothesis is that there is no difference in the occurrence of trauma-induced coagulopathy between the standard and plasma groups. The alternative hypothesis is that there is a difference between the two groups.
- Objectives
- Principal
  - To show the effectiveness of FLYP administered prehospital during the management of hemorrhagic shock of traumatic origin, on the occurrence or treatment of post-traumatic coagulopathy.
- Secondary
  - Feasibility of FLYP administration in the prehospital setting
  - Show that FLYP improves fibrinogen levels
  - To show that the contribution of FLYP decreases the transfusion needs (Red Blood Cells, plasma, coagulation factors, platelets)
  - To show that the contribution of FLYP decreases the length of stay in intensive care
  - Compare mortality between the two groups until D30
  - Validate the safety of plasma transfusion in the prehospital setting.

## 16.3 Trial design

- The trial is a Multicenter, randomized, open-label controlled study. From the prehospital phase, the FLYP+ group receives the reference treatment, both symptomatic and etiological, as well as the administration of FLYP. The control group (FLYP - group) receives the reference treatment, both symptomatic and etiological, but no administration of FLYP, which is replaced by saline.

## 16.4 Randomization

- Randomization assigns either the FLYP treatment combined with the usual treatment or the usual treatment alone.
- CTSA randomizes study treatments in blocks of 2 boxes (one with FLYP, one without).

- The treatment boxes are numbered in ascending order from 1 to 140.
- The CTSA and the EFS will distribute the numbered boxes to the prehospital investigating centers as they are included (at the request of the centers) and by ascending order from 1 to 140. Only one pack will be available in each ALS vehicle at the time of patient management.
- The randomization list will be maintained by CTSA and SSA.

**16.5 Sample size**

- A previous observational study in a military setting (reference 18 in the study protocol) in patients with traumatic hemorrhage of "before-after" FLYP administration with Prothrombin Time (PT) as endpoint showed a significant improvement in PT after FLYP. A total of 124 patients were required to observe a statistically significant difference.
- After the change in the primary endpoint, the sample size was revised and recalculated. Given that we could not increase the number of patients due to logistical, organizational, and financial reasons, we calculated the power we could achieve with the same number of patients.
- We calculated that a sample size of 30 patients with coagulopathy would be appropriate in each group to detect a mean difference of 0·3* in international normalized ratio values at hospital admission, assuming a standard deviation of 0.4, with a two-sided type I error of 0.05 and a power of 80%. Assuming an incidence of coagulopathy of 50% † , this calculation results in 60 patients per group. With an a priori assumed loss of 10% because of lack of informed consent, we round the size of each group to 70 for a total of 140 patients.
- The concession to the original calculation is essentially a 0.1 increase in the standard deviation and a power loss from 90% to 80%.
- We anticipate calculating the number of subjects to compare two medians in case the primary endpoint's distribution is not Gaussian. Indeed, let's consider a median difference of 0·3 in international normalized ratio values instead of a mean difference. The sample size should be 25 patients with coagulopathy in each group, under the same assumptions of alpha (0·05) and beta (20%) risk.

*This intervention effect is only based on one thesis, and is not based on previous valid studies because we could not identify such studies in the literature. We pragmatically chose the anticipated effects based on one observational study and clinical judgment. Therefore, a difference of 0·3 observed in a time frame as short as that of a prehospital intervention seemed to be the minimum clinically significant difference relevant to us.

† This incidence is based on an international normalized ratio level threshold of 1·2 and is partially explained by a significant increase in severity in injured patients when the study protocol was written.

**Framework**

- The outcomes are testing for the superiority hypothesis.
- There are no plans to conduct an interim analysis.

**Timing of final analysis**

- All outcomes will be analyzed collectively when the required number of inclusions across the standard care vs. plasma arms, and follow-up of 30 days, have been reached.

**Timing of outcome assessments**

- The schedule of study procedures is given in paragraph 3·3·5 of the protocol.

The start time for each calculation is the participant's date of inclusion.

**Confidence intervals and P values**

- All statistical tests will be 2-sided and performed using a 5% significance level and 95% two-sided confidence intervals.
- No adjustment for multiplicity is planned
- 95% two-sided confidence intervals

**Adherence and Protocol deviations**

- Protocol deviations are defined as:
  - Enrollment without meeting inclusion criteria
  - Incomplete or lack of patient consent
- The number (and percentage) of patients with major and minor protocol deviations will be summarized by treatment group, with details on the type of deviation. Protocol deviations will be screened for in the intention-to-treat population.

**Analysis populations**

- The people analyzed will be:
  - for the safety outcome: all the randomized patients (intention-to-treat population).
  - for the primary and secondary outcomes: all the randomized patients except those who have an exclusion criterion (modified intention-to-treat population)
  - for a sensibility analysis for the primary outcome: all the randomized patients except those who have an exclusion criterion and those who finally have no bleeding after hospital assessment (per-protocol analysis)

## 16.6 Trial Population

- Screening data
  - The number of patients screened will be reported for each investigating center. The flow chart will note the total for all centers
- Eligibility
  - The Consort Flow chart will report the number of patients eligible and not eligible due to an exclusion criterion (paragraph 4· in the protocol)
- Recruitment, Withdrawal and Follow-up
  - The "CONSORT" diagram will report the number of people screened, eligible, consented, randomized, receiving their allocated treatment.
  - The level of consent withdrawal will be tabulated as consent to continue follow-up and data collection
  - Timing and number of patients lost to follow-up will be presented in a Kaplan-Meier Diagram.
  - The numbers of losses to follow-up will be summarized per treatment arm
- Baseline patient characteristics
  - Patients will be described with respect to age, sex, comorbidities, type of injury, mechanism of injury, anatomic localization of trauma, time response of advanced life support teams, vital status at the point of injury (blood pressure, heart rate, shock-index, oxygen saturation),

prehospital biological measures (Prothrombin time and INR value, hemoglobin and lactic acid concentrations), body temperature, basic life support therapeutics, advanced life support therapeutics, cumulated crystalloid volume, cumulated plasma volume, prehospital red cell units transfused, vital signs on arrival at the intensive care unit (blood pressure, heart rate, shock-index, injury severity score, traumatic brain injury confirmed with CT scan, laboratory values - hemoglobin, platelets, lactic acid, base excess)

- The description will be reported separately for the two randomized groups. Categorical data will be summarized by numbers and percentages. Continuous data will be summarized by mean, SD, and range if data are Gaussian, and median, IQR, and range if data are skewed. Tests of statistical significance will not be undertaken for baseline. Instead, the clinical importance of any imbalance will be noted.

## 16.7 Outcome definitions

- The primary endpoint is the international normalized ratio (INR) at hospital arrival.
- Secondary outcomes (prehospital and hospital phases)
- The delta-INR value between the prehospital INR and the INR at hospital arrival. The Point of Care Device Coaguchek® will measure these two INR values
- The change in fibrinogen between the fibrinogen on hospital admission and the fibrinogen initially collected in the prehospital setting.
- Number of RBCs, platelet concentrates, fibrinogen, coagulation factors, plasma, administered in the first 24 and 48 hours.
- The total length of stay in intensive care
- Survival in the first 30 days.
- Collection of technical and logistical difficulties encountered before, during and after the administration of FLYP.

**Analysis methods**

**First endpoint**

- For the primary outcome analysis, INR at hospital admission will be considered to have been performed at a comparable time in each patient and will be compared across the two arms with the Mann–Whitney U test. If the initial characteristics of patients are unbalanced between the two groups after randomization, an adjusted analysis will be considered with a regression method. When appropriate, both adjusted and unadjusted results will be reported.

**Secondary endpoints**

- Comparison of fibrinogen changes by group: paired t-test, analysis of covariance
- Comparison of the volumes of blood loss, volumes infused or transfused, the quantity of amines used between the two groups (Chi2, Fisher's exact, Student's, Mann-Whitney tests).
- Comparison of the INR differential, delta INR : [INR at hospital admission - initial INR at the point of injury] between the two groups

- We will compare the Delta-INR values between the plasma and control groups using a linear mixed model with an independent covariance matrix, considering study group, time, and Injury Severity Score levels as fixed effects and incorporating a random intercept.
- Comparison of the length of stay in intensive care between the 2 groups : Student's t test, Mann-Whitney test.
- Number of days of hospitalization: Student's t-test, Mann-Whitney test
- Survival analysis over the first 30 days: Kaplan Meier graphic, log-rank test.
- Feasibility endpoint : listing of technical and logistical difficulties in plasma administration , number ad percentage of patients, number and percentage of plasma vials.
- The analysis will all take into account a possible center effect.
- Safety Endpoint is the occurrence of any of the following adverse events: thrombosis, allergic symptoms, transfusion-related acute lung injury, and transfusion-associated circulatory overload. The traceability of plasma, including prescription, delivery, and follow-up procedures, will be assessed using the plasma follow-up sheet from the French Military Blood Institute. The Health Defence Service will realize the qualitative and quantitative analysis of Adverse Events. There is no formal statistical test preplanned
- If the initial characteristics of patients are unbalanced between the two groups after randomization, an adjusted analysis will be considered with a regression method. Where appropriate, both adjusted and unadjusted results will be reported.
- The normality assumption will be examined graphically and estimated with the Shapiro-Wilk test. If the normality hypothesis is not verified, the estimator of the variable will be the median and the interquartile range.
- If normality is not assumed, the estimators for the continuous variables will be the median and the interquartile range. In addition, continuous variables will be transformed into categorical variables.
- For the primary outcome in the modified intention-to-treat population, imputed data will be analyzed and compared with a complete-case analysis. If there is no difference, the imputed data will be presented.
- For the following non-prespecified subgroups, we will report the effect size on the INR Value at hospital arrival: type of injury (blunt vs. penetrating), pelvic injury, prehospital vasopressor use, prehospital intubation, brain injury with contusions or bleeding on computed tomography scan, massive transfusion, and death up to 24 hours.
- We will express effect sizes as median differences (95% CIs) for continuous variables and as risk ratios (95% CIs) for categorical variables.

### 16.8 Missing data

- Missing data will be processed by multiple imputation.
- Invalid data will be considered missing.
- Multiple imputation procedures will be performed on the data of patients involved in the modified intention-to-treat analysis (i.e., full ITT except for the patients who have an exclusion criterion). We will address the issue of missing data using the state-of-the-art multiple imputation techniques, including all analysis variables, under the assumption that missing values are missing at random. We will use the MI impute chained command, generate a number of imputed datasets depending on the fraction of missing information. Analyses run on each dataset will be pooled according to Rubin's rules. The multiple imputation diagnostic plots will be reported for the variables with the highest missing rate. We will compare the distributions of the observed, imputed, and completed values. We will use the kernel- Epanechnikov density

estimator for continuous variables and a histogram for categorical variables. We will use Kolmogorov- Smirnov statistics to compare the observed with the imputed distributions of each continuous variable in imputed variables. The null hypothesis (i.e., observed and imputed data follow the same distribution) is rejected at P<0·05. The imputed results will be reported as the main results because they are similar to the complete case analysis estimates.

### 16.9 Additional analyses

We will provide :

- a multinomial logistic regression model to assess the relationship between the INR level and the plasma-to-crystalloid volume ratio.
- The main surgical procedures by group in the first 24 hours in the modified intention to treat analysis.
- Univariate logistic regression models for the association between INR and other covariates.

**Harms**

- The number of patients experiencing AE will be presented for each treatment arm.
- For each patient, only the maximum severity experienced of each type of AE will be displayed. The number of occurrences of each AE will also be presented for each treatment arm. No statistical testing will be undertaken

**Statistical software**

- We will use STATA® software version 14·0 or an updated version for Windows (STATACORP LLC, College Station, Texas, USA)
