# Supplementary material for: Prehospital Lyophilized Plasma Transfusion for Trauma-Induced Coagulopathy in Patients at Risk for Hemorrhagic Shock: A Randomized Clinical Trial
Source: JAMA Netw Open. 2022 Jul 26;5(7):e2223619. doi: 10.1001/jamanetworkopen.2022.23619 (PMC9327575; doi:10.1001/jamanetworkopen.2022.23619)
Supplement: Supplement 3. — Nonauthor Collaborators [file jamanetwopen-e2223619-s003.pdf]

Supplemental Online Content: Nonauthor Collaborators
*First name, last name, and suffix (if applicable) are required and will appear in PubMed.

*Group Name(s): Prehospital Lyophilized Plasma (PREHO-PLYO) Study Group							
***First Name and Middle Initial(s)**	***Last Name**	***Suffix (eg, Jr, III)**	Academic Degrees	Institution	Location (city, state/province, country)	Role or Contribution, eg, chair, principal investigator	Group (if more than 1 Group listed in the byline) and/or Subgroup (eg, Steering Committee)
Souha	Albinni		MD	Necker-Enfants malades Hospital	Paris, France	Collaborator	
Arié	Attias		MD	Henri Mondor University Hospital	Creteil, France	Collaborator	
Deborah	Benchetritt		MD	Pitié-Salpêtrière University Hospital	Paris, France	Collaborator	
Laura	Benichou		MD	la Cavale Blanche University Hospital	Brest, France	Collaborator	
René	Bihannic		PharmD	Paris Fire Brigade, Emergency Medici	Paris, France	Collaborator	
Stéphane	Boizat		MD	Paris Fire Brigade, Emergency Medici	Paris, France	Collaborator	
Philippe	Boutinaud			Paris Fire Brigade	Paris, France	Collaborator	
Alexandra	Calinet		PharmD	DFRI, French Military Health Service	Paris, France	Collaborator	
Laurence	Camoin-Jau		MDPhD	Timone University Hospital	Marseille, France	Collaborator	
Valérie	Cerro		CRA	Édouard-Herriot University Hospital	Lyon, France	Collaborator	
Eric	Cesareo		MD	Édouard-Herriot University Hospital	Lyon, France	Collaborator	
Noureddine	Chahir			la Cavale Blanche University Hospital	Brest, France	Collaborator	
Carine	Chassery			Édouard-Herriot University Hospital	Lyon, France	Collaborator	
Charlotte	Chollet		MD	Henri Mondor University Hospital	Creteil, France	Collaborator	
Anouk	Choubard		Nurse	Édouard-Herriot University Hospital	Lyon, France	Collaborator	
Gaëlle	Clavere		CRA	Annecy-Genevois Hospital	Annecy, France	Collaborator	
Benoit	Clavier			Percy Military Teaching Hospital	Clamart, France	Collaborator	
Henri	Courtade			F. Mitterrand Hospital	Pau, France	Collaborator	
Séverine	Creppy			Édouard-Herriot University Hospital	Lyon, France	Collaborator	
Jean-Stéphane	David		MDPhD	Lyon Sud University Hospital	Lyon, France	Collaborator	
Emmanuelle	De Raucourt		MDPhD	Beaujon University Hospital	Clichy, France	Collaborator	
Sophie	Debord			Édouard-Herriot University Hospital	Lyon, France	Collaborator	
Josée	Delort			Pitié-Salpêtrière University Hospital	Paris, France	Collaborator	
Christine	Deruaz-Cunsolo			Annecy-Genevois Hospital	Annecy, France	Collaborator	
Isabelle	Dettori			Timone University Hospital	Marseille, France	Collaborator	
Marion	Dhers		Nurse	Édouard-Herriot University Hospital	Lyon, France	Collaborator	
Patricia	Dias		CRA	la Cavale Blanche University Hospital	Brest, France	Collaborator	
Maxime	Diaz		MD	Beaujon University Hospital	Clichy, France	Collaborator	
Sophie	Dieuset			la Cavale Blanche University Hospital	Brest, France	Collaborator	
Pierre-Yves	Dubien		MDPhD	Édouard-Herriot University Hospital	Lyon, France	Collaborator	

Supplemental Online Content: Nonauthor Collaborators
*First name, last name, and suffix (if applicable) are required and will appear in PubMed.

***First Name and Middle Initial(s)**	***Last Name**	***Suffix (eg, Jr, III)**	Academic Degrees	Institution	Location (city, state/province, country)	Role or Contribution, eg, chair, principal investigator	Group (if more than 1 Group listed in the byline) and/or Subgroup (eg, Steering Committee)
François-Xavier	Duchateau		MD	Beaujon University Hospital	Clichy, France	Collaborator	
Jacques	Duranteau		MDPhD	Bicêtre University Hospital	Kremlin-Bicêtre, France	Collaborator	
Charlotte	Fiot			Annecy-Genevois Hospital	Annecy, France	Collaborator	
Bernard	Flocard		MDPhD	Édouard-Herriot University Hospital	Lyon, France	Collaborator	
Vincent	Foissaud			Percy Military Teaching Hospital	Clamart, France	Collaborator	
Marc	Fournier		MD	Timone University Hospital	Marseille, France	Collaborator	
Anne	Francois			European Georges Pompidou Hospita	Paris, France	Collaborator	
Gilles	Gaget			Édouard-Herriot University Hospital	Lyon, France	Collaborator	
Benjamin	Garnier		MD	la Cavale Blanche University Hospital	Brest, France	Collaborator	
Jean-Louis	Gaste		PharmD	Marseille Naval Fire Battalion	Marseille, France	Collaborator	
Olivier	Grimault			la Cavale Blanche University Hospital	Brest, France	Collaborator	
Pierre-Yves	Gueugniaud		MDPhD	Édouard-Herriot University Hospital	Lyon, France	Collaborator	
Zakia	Idir			Department Clinical Research of Dev	Paris, France	Collaborator	
Jerome	Burnichon		Nurse	Annecy-Genevois Hospital	Annecy, France	Collaborator	
Didier	Journois		MDPhD	European Georges Pompidou Hospita	Paris, France	Collaborator	
Florence	Blanc-Jouvan			Annecy-Genevois Hospital	Annecy, France	Collaborator	
Francois	Kerbaul		MDPhD	Timone University Hospital	Marseille, France	Collaborator	
Isabelle	Klein		MD	Paris Fire Brigade, Emergency Medici	Paris, France	Collaborator	
Blandine	Lafitte		CRA	Annecy-Genevois Hospital	Annecy, France	Collaborator	
Olivier	Langeron		MDPhD	Henri Mondor University Hospital	Paris, France	Collaborator	
Bernard	Lassale			Sainte-Marguerite Hospital	Marseille, France	Collaborator	
Marie	Lebouc			Annecy-Genevois Hospital	Annecy, France	Collaborator	
Eric	Lecarpentier		MD	Henri Mondor University Hospital	Creteil, France	Collaborator	
Stephanie	Lejeune			Annecy-Genevois Hospital	Annecy, France	Collaborator	
Mickaël	Lemaire		PharmD	Paris Fire Brigade, Emergency Medici	Paris, France	Collaborator	
Catherine	Leniger			la Cavale Blanche University Hospital	Brest, France	Collaborator	
Marc	Leone		MDPhD	North Hospital	Marseille, France	Collaborator	
Claudie	Leostic			la Cavale Blanche University Hospital	Brest, France	Collaborator	
Chantal	Lutomski		HCE	Percy Military Teaching Hospital	Clamart, France	Collaborator	
Marianne	Sailliol		MD	F. Mitterrand Hospital	Pau, France	Collaborator	
Claude-Denis	Martin		MDPhD	North Hospital	Marseille, France	Collaborator	
Catherine	Matheron		MD	Henri Mondor University Hospital	Creteil, France	Collaborator	
Jacques	Mathieu			DFRI French Military Health Service	Paris, France	Collaborator	

Supplemental Online Content: Nonauthor Collaborators
*First name, last name, and suffix (if applicable) are required and will appear in PubMed.

***First Name and Middle Initial(s)**	***Last Name**	***Suffix (eg, Jr, III)**	Academic Degrees	Institution	Location (city, state/province, country)	Role or Contribution, eg, chair, principal investigator	Group (if more than 1 Group listed in the byline) and/or Subgroup (eg, Steering Committee)
Anne-Christine	Mendes			Percy Military Teaching Hospital	Clamart, France	Collaborator	
Romain	Mermillod-Blondin			Annecy-Genevois Hospital	Annecy, France	Collaborator	
Arthur	Neuschwander		MD	European Georges Pompidou Hospita	Paris, France	Collaborator	
Nathalie	Oueidat		PharmD	Pitié-Salpêtrière University Hospital	Paris, France	Collaborator	
Mehdi	Ould-Ahmed		MDPhD	Begin Military Teaching Hospital	Saint-Mandé, France	Collaborator	
Yves	Ozier		MDPhD	la Cavale Blanche University Hospital	Brest, France	Collaborator	
Catherine	Paugam		MDPhD	Beaujon University Hospital	Clichy, France	Collaborator	
Franck	Peduzzi		MD	Marseille Naval Fire Battalion	Marseille, France	Collaborator	
Alexandre	Petermann		Secretary	Paris Fire Brigade, Emergency Medici	Paris, France	Collaborator	
Eric	Peytel		MDPhD	Laveran Military Teaching Hospital	Marseille, France	Collaborator	
Muriel	Picou-Leblanc			Annecy-Genevois Hospital	Annecy, France	Collaborator	
David	Pinero			Édouard-Herriot University Hospital	Lyon, France	Collaborator	
Patrick	Plaisance		MDPhD	Lariboisière Hospital	Paris, France	Collaborator	
Michel	Raba			Lyon Sud University Hospital	Lyon, France	Collaborator	
Damien	Ricard		MDPhD	Percy Military Teaching Hospital	Clamart, France	Collaborator	
Isabelle	Romanacce			Lariboisière Hospital	Paris, France	Collaborator	
Thomas	Rossignol			F. Mitterrand Hospital	Pau, France	Collaborator	
Christine	Rougelin-Clapasson			Timone University Hospital	Marseille, France	Collaborator	
Amelie	Saint-Paul			Édouard-Herriot University Hospital	Lyon, France	Collaborator	
Salim	Idri			Beaujon University Hospital	Clichy, France	Collaborator	
Christine	Scotto		Nurse	North Hospital	Marseille, France	Collaborator	
Laurence	Senent		MD	Timone University Hospital	Marseille, France	Collaborator	
David	Smadja		MDPhD	European Georges Pompidou Hospita	Paris, France	Collaborator	
Karim	Tazarourte		MDPhD	Édouard-Herriot University Hospital	Lyon, France	Collaborator	
Francois	Topin		MD	Marseille Naval Fire Battalion	Marseille, France	Collaborator	
Catherine	Trichet		MD	Beaujon University Hospital	Clichy, France	Collaborator	
Alexia	Vocel			Annecy-Genevois Hospital	Annecy, France	Collaborator	
