# Supplementary material for: Prehospital Lyophilized Plasma Transfusion for Trauma-Induced Coagulopathy in Patients at Risk for Hemorrhagic Shock: A Randomized Clinical Trial
Source: JAMA Netw Open. 2022 Jul 26;5(7):e2223619. doi: 10.1001/jamanetworkopen.2022.23619 (PMC9327575; doi:10.1001/jamanetworkopen.2022.23619)
Supplement: Supplement 4. — Data Sharing Statement [file jamanetwopen-e2223619-s004.pdf]

Data Sharing Statement

Jost. Prehospital Lyophilized Plasma Transfusion for Trauma-Induced Coagulopathy in Patients at Risk for Hemorrhagic Shock. *JAMA Netw Open*. Published July 26, 2022. doi:10.1001/jamanetworkopen.2022.23619

Data
Data available: No
